# Comparative Assessment of the New PDE7 Inhibitor – GRMS-55 and Lisofylline in Animal Models of Immune-Related Disorders: A PK/PD Modeling Approach

**DOI:** 10.1007/s11095-019-2727-z

**Published:** 2020-01-02

**Authors:** Artur Świerczek, Krzysztof Pociecha, Marietta Ślusarczyk, Grażyna Chłoń-Rzepa, Sebastian Baś, Jacek Mlynarski, Krzysztof Więckowski, Monika Zadrożna, Barbara Nowak, Elżbieta Wyska

**Affiliations:** 1 Department of Pharmacokinetics and Physical Pharmacy, Jagiellonian University Medical College, 9 Medyczna Street, 30-688 Kraków, Poland; 2 Department of Medicinal Chemistry, Jagiellonian University Medical College, 9 Medyczna Street, 30-688 Kraków, Poland; 3 Faculty of Chemistry, Jagiellonian University, Gronostajowa 2, 30-387 Kraków, Poland; 4 Present Address: Institute of Organic Chemistry, Polish Academy of Sciences, Kasprzaka 44/52, 01-224 Warsaw, Poland; 5 Department of Organic Chemistry, Jagiellonian University Medical College, 9 Medyczna Street, 30-688 Kraków, Poland; 6 Department of Cytobiology, Jagiellonian University Medical College, 9 Medyczna Street, 30-688 Kraków, Poland

**Keywords:** Phosphodiesterase inhibitors, disease progression modeling, collagen-induced arthritis, autoimmune hepatitis, endotoxemia

## Abstract

**Purpose:**

This study aimed to assess the activity of two phosphodiesterase (PDE) inhibitors, namely GRMS-55 and racemic lisofylline ((±)-LSF)) *in vitro* and in animal models of immune-mediated disorders.

**Methods:**

Inhibition of human recombinant (hr)PDEs and TNF-alpha release from LPS-stimulated whole rat blood by the studied compounds were assessed *in vitro*. LPS-induced endotoxemia, concanavalin A (ConA)-induced hepatitis, and collagen-induced arthritis (CIA) animal models were used for *in vivo* evaluation. The potency of the investigated compounds was evaluated using PK/PD and PK/PD/disease progression modeling.

**Results:**

GRMS-55 is a potent hrPDE7A and hrPDE1B inhibitor, while (±)-LSF most strongly inhibits hrPDE3A and hrPDE4B. GRMS-55 decreased TNF-alpha levels *in vivo* and CIA progression with *IC*_*50*_ of 1.06 and 0.26 mg/L, while (±)-LSF with *IC*_*50*_ of 5.80 and 1.06 mg/L, respectively. Moreover, GRMS-55 significantly ameliorated symptoms of ConA-induced hepatitis.

**Conclusions:**

PDE4B but not PDE4D inhibition appears to be mainly engaged in anti-inflammatory activity of the studied compounds. GRMS-55 and (±)-LSF seem to be promising candidates for future studies on the treatment of immune-related diseases. The developed PK/PD models may be used to assess the anti-inflammatory and anti-arthritic potency of new compounds for the treatment of rheumatoid arthritis and other inflammatory disorders.

**Electronic supplementary material:**

The online version of this article (10.1007/s11095-019-2727-z) contains supplementary material, which is available to authorized users.

## Introduction

Phosphodiesterases (PDEs) are enzymes that hydrolyze the second messengers – 3′,5′-cyclic adenosine monophosphate (cAMP) and 3′,5′-cyclic guanosine monophosphate (cGMP). Eleven types of this enzyme have been described to date. Some PDEs, namely PDE4, 7, and 8 are specific for cAMP (1–3), while other, such as PDE5, 6, and 9 for cGMP (4–6). The remaining family members, i.e., PDE1, 2, 3, 10, and 11 exhibit dual cAMP/cGMP specificity (5,7,8). Due to the ubiquitous expression of PDEs in the mammalian organisms, PDE inhibitors have emerged as a large group of biologically active compounds that may be used for the treatment of many disorders. PDE4 and PDE7 are expressed in immune cells, thus, in recent years, these types of PDE have become a target for the treatment of immune-related diseases, such as inflammatory and autoimmune disorders (2,9). It is well known that T cells and increased levels of cytokines, such as TNF-α are involved in the development of these conditions that include, among the others, rheumatoid arthritis (RA), autoimmune hepatitis (AIH), or sepsis. These enzymes by affecting cAMP levels not only contribute to the regulation of immune response with respect to cytokine/chemokine production but also control lymphocyte proliferation. It has been demonstrated that an increase in intracellular cAMP levels causes a repression of immune responses. In contrast, a reduction of cAMP concentrations may exert an immunostimulatory effect (10). TNF-α is considered a key cytokine in the development and course of RA, AIH, as well as sepsis (11–13). Thus, anti-TNF-α therapies are gaining a growing interest as a treatment option of these diseases. Selective PDE4 and PDE7 inhibitors have been shown to decrease the release of pro-inflammatory mediators, such as TNF-α and many other from stimulated immune cells *in vitro* as well as *in vivo* in various animal models of inflammatory and autoimmune disorders (2). Interestingly, the results of some pre-clinical studies indicate that an inhibition of PDE4B subtype is mainly engaged in the anti-inflammatory activity of selective PDE4 inhibitors (1,14), while a PDE4D blockage leads to the undesirable effects, such as nausea and emesis (15).

Therefore, it seems possible that the use of PDE inhibitors that act on PDE4B and PDE7 and do not demonstrate inhibitory activity against PDE4D may offer a better therapeutic option compared to administration of potent pan-PDE4 inhibitors due to a widened therapeutic window. Such compounds should retain anti-inflammatory activity via an inhibition of PDE4B and at the same time be devoid of adverse effects resulting from PDE4D inhibition. Moreover, a PDE7 inhibitory component, which may contribute to the immunoregulatory activity of such compounds, could demonstrate an additional therapeutic advantage in the treatment of inflammatory and autoimmune disorders.

The aim of this study was to compare the pharmacological activity of two PDE inhibitors, namely a new 1,3-dimethylpurine-2,6-dione derivative: 4-(1,3-Dimethyl-2,6-dioxo-8-(phenethylamino)-2,3,6,7-tetrahydro-1*H*-purin-7-yl)-N′-(2-hydroxybenzylidene)butanehydrazide (GRMS-55) and racemic lisofylline ((±)-LSF) to address the issues raised above. GRMS-55 has been chosen from the library of newly designed and synthesized compounds as a moderate inhibitor of PDE4B and a strong inhibitor of PDE7A (16). (±)-LSF is a drug candidate with a moderate PDE4B inhibitory activity tested earlier in pre-clinical and clinical studies as a potential drug for the treatment of inflammatory and autoimmune disorders (9,17). In *in vitro* experiments, the selectivity of the test compounds against various subtypes of human recombinant (hr)PDEs and their ability to inhibit TNF-α release from whole rat blood stimulated by lipopolysaccharide (LPS) were investigated. Both compounds were subsequently evaluated in *in vivo* models of inflammatory and autoimmune disorders, namely in the mice model of AIH induced by an intravenous (IV) injection of concanavalin A (ConA), in rat endotoxemia evoked by IV administration of bacterial LPS, which is an animal model of sepsis, and finally, in the collagen-induced arthritis (CIA) in rats that is an accepted and well recognized model of RA. PK/PD and PK/PD/disease (DIS) progression models have been developed and utilized in this study as the tools enabling the quantitative assessment of activity of the investigated and reference compounds.

## Materials and Methods

### Reagents and Compounds

Pentoxifylline (PTX), dimethylsulfoxide (DMSO), polyethylene glycol (PEG) 400, LPS (*E. coli* 055:B5 serotype), incomplete Freund’s adjuvant (IFA), and RPMI 1640 medium were purchased from Sigma-Aldrich (Germany). ConA was purchased from Santa Cruz Biotechnology (USA). Porcine collagen type II (2 mg mL^−1^ in 0.05 M acetic acid) was obtained from Chondrex, Inc. (USA). Vinpocetine, EHNA, milrinone, zaprinast, and papaverine were purchased from Cayman Chemical (USA). (±)-LSF was obtained from the Department of Organic Chemistry, Faculty of Chemistry, Jagiellonian University (Krakow, Poland). GRMS-55 and 4-(8-((Furan-2-ylmethyl)amino)-1,3-dimethyl-2,6-dioxo-2,3,6,7-tetrahydro-1*H*-purin-7-yl)-N′-(2-hydroxybenzylidene)butanehydrazide were obtained from the Department of Medicinal Chemistry and rolipram from the Department of Organic Chemistry, Faculty of Pharmacy, Jagiellonian University Medical College (Krakow, Poland). Temazepam was a gift from Polfa (Poland). Other reagents and solvents were of HPLC or analytical reagent grade and were purchased from Merck (Germany).

### Animals

10–12-week-old female Lewis rats were used in the CIA experiment and in pharmacokinetic studies and male 8–12-week-old male Wistar rats were utilized to evoke LPS-induced endotoxemia model and in pharmacokinetic studies. Female BALB/c mice weighing 20 ± 3 g were used in ConA-induced hepatitis model. Lewis rats and BALB/c mice were purchased from the Mossakowski Medical Research Center Polish Academy of Sciences (Warsaw, Poland), and Wistar rats were from the Animal Facility at the Faculty of Pharmacy, Jagiellonian University Medical College (Krakow, Poland). Animals were housed in conditions of the constant temperature (21°C) with a 12:12 h light–dark cycle with free access to food and water. All animal procedures were approved by the First Local Ethical Committee on Animal Testing at the Jagiellonian University. All applicable international, national, and institutional guidelines for the care and use of animals were followed.

### PDE Assay

Inhibitory activity of the investigated and reference compounds against various subtypes of PDEs was analyzed using the PDE-Glo Phosphodiesterase Assay following the manufacturer’s instruction (Promega Corp., USA). A brief description of this procedure was presented in the supplementary materials. The values of *IC*_*50*_, *I*_*max*_, and *γ* parameters of the investigated and reference compounds were estimated using non-linear regression in Phoenix WinNonlin v. 7.0. Each sample was performed in quadruplicate and the relative activity of each sample was calculated as $E=\frac{sample\ activity}{control\ activity}\cdotp 100\%$. Subsequently, the following equation was fitted to the effect-concentration data:

1 $$ E={E}_0-{I}_{max}\cdotp \left(\frac{C^{\gamma }}{C^{\gamma }+{IC_{50}}^{\gamma }}\right) $$

where *E*_0_ is a baseline relative activity of each enzyme in the absence of a studied compound (fixed to 100%). *I*_*max*_ is the maximal inhibitory activity of a compound, *C* is the concentration of a compound, *IC*_50_ is the concentration of a compound that produces 50% of *I*_*max*_, and γ is the Hill coefficient.

### Stimulation of Whole Rat Blood

Stimulation of whole rat blood by LPS was performed on 96-well plates. The inhibitory activities of GRMS-55, (±)-LSF, and reference compounds, i.e., rolipram and PTX were tested. Contents of each well (200 μL) consisted of heparinized rat blood containing 10 U heparin mL^−1^ diluted with RPMI-1640 medium at a ratio of 1:1 (*v*/v). All compounds were dissolved in DMSO and added to blood (the final DMSO concentration in blood was 0.5%) 15 min prior to the addition of LPS dissolved in RPMI-1640 medium to achieve the LPS concentration of 10 mg L^−1^. Both, LPS and DMSO were also added to control wells at the same concentrations. Additional control samples (for testing baseline TNF-α levels in blood) consisted of DMSO (0.5%) without LPS. The incubation was performed at 37°C in a humidified atmosphere of 5% CO_2_ in an MCO-5 AC-PE incubator (PHCbi, Japan). After 6 h of incubation, the plates were centrifuged for 10 min using PlateFuge MicroCentrifuge (Benchmark Scientific, USA). The individual plasma samples were harvested and stored at −80°C until analysis.

### Induction of Hepatitis

ConA was dissolved in an appropriate volume of saline for injections to obtain a concentration of 2 mg mL^−1^. Each of the investigated compounds was dissolved in a vehicle composed of DMSO and PEG 400 mixed at a ratio of 1:9 (*v*/v). Thirty minutes before ConA administration, mice were injected intraperitoneally (IP) with the investigated compounds dissolved in the vehicle at a dose of 50 mg kg^−1^ or with the corresponding volume of vehicle alone in the case of control group. ConA was administered by IV route at a dose of 20 mg kg^−1^ and 8 h later mice were sacrificed. Blood samples were harvested from each individual and allowed to clot for 30 min at room temperature. Subsequently, serum was separated by centrifugation (3000 x g, 10 min), collected, and stored at −80°C until analysis.

### Induction of Endotoxemia

Male Wistar rats were implanted with catheters (SAI Infusion Technologies, USA) into the jugular vein and after a two-day recovery period they were administered with LPS dissolved in saline at a dose of 1 mg kg ^−1^ into the tail vein simultaneously with one of the investigated compounds. GRMS-55 and rolipram were dissolved in vehicle composed of 10% DMSO in saline and given at doses of 20 and 5 mg kg^−1^, respectively, while (±)-LSF and PTX were dissolved in saline and were administered at two doses of 40 and 80 mg kg^−1^. The doses of the compounds were selected based on the literature data or results of our previous experiments. Each control group received LPS at the same IV dose and an appropriate volume of corresponding vehicle. Blood samples were collected via catheters into polypropylene heparinized snap-cap tubes at 0, 15, 30, 60, 90, 120, and 180 min following (±)-LSF and PTX dosing and at 30, 60, 90, 120, and 180 min after GRMS-55 and rolipram administration and centrifuged at 3000 x g for 10 min. The plasma samples were collected and stored at −80°C until analysis.

### Induction of Arthritis

The emulsion of collagen was prepared according to the *Protocol for the Successful Induction of Collagen-Induced Arthritis (CIA) in Rats* (Chondrex Inc., 2015). Porcine type II collagen dissolved in 0.05 M acetic acid was emulsified with incomplete Freund’s adiuvant (Sigma Aldrich, Germany) using an Omni TH Homogenizer (Omni International, Inc., USA) consisting of a blade with 5 mm diameter. The collagen solution was previously mixed with IFA at a low speed for 1 min and then at the highest speed for 2 min. Then emulsion was being cooled for 5 min in an ice water bath. The emulsification process was repeated 3 times to obtain a stiff emulsion. The emulsion was then transferred into a Hamilton glass syringe, kept at 4°C, and used within 2 h.

At a day 0 of the experiment animals were subcutaneously (SC) injected at the base of the tail with 200 μL of the emulsion using a 27 gauge × 5/8″ needle and Hamilton syringe. At day 7 after the initial immunization, a booster injection with 100 μL of emulsion was performed. Ninety % of rats developed symptoms of disease (paw swelling >150% of day 0) at day 20 of the experiment. At this day, diseased animals were randomly divided into five groups (*n* = 6). Two investigated groups received IP GRMS-55 at a dose of 50 mg kg^−1^ or rolipram at a dose of 10 mg kg^−1^ once daily, dissolved in a vehicle composed of 90/10 (*v*/v) of PEG 400 and DMSO. One investigated group received (±)-LSF dissolved in saline at an SC dose of 80 mg kg^−1^ once daily. The corresponding control groups were administered with appropriate volumes of vehicles IP or SC. At day 40 animals were sacrificed. Hind paws of rats were taken and immersed into 4% buffered formalin solution for a subsequent histological analysis. Blood samples were collected and allowed to clot for 20 min and then centrifuged at 2000 x g for 15 min. Serum samples were collected and frozen at -80°C.

#### Measurement of Edema

Paw edema of arthritic rats was assessed as swelling of the rat hind paws. Measurements of paws were performed using a digital caliper according to the procedure described by Earp *et al.* (18). Each hind leg was measured at two sections, i.e., paw and ankle, and each section was measured at two places (side-to-side and top-to-bottom). The area of each section (elliptical in shape) was calculated from the following equation:

2 $$ Area=\pi \cdotp \frac{\mathrm{a}\cdotp \mathrm{b}}{4} $$

where (a) is side-to-side diameter of the ellipse and (b) is top-to-bottom diameter of the ellipse. Paw and ankle section areas of each leg were subsequently added to obtain one overall measure for the rat hind paw (*Area*_*overall*_). Each of these measures was normalized to the same hind paw area measured at day 0 of the experiment (*Area*_*zero*_) to reduce variation in the disease progression resulting from differences in paw size of each rat. Relative paw swelling (*Paw*) was calculated according to the equation:

3 $$ Paw=\frac{Area_{overall}}{Area_{zero}} $$

Paw size was measured at days 0, 5, 7, 11, 15, 18, 20, 22, 26, 30, 33, and 36 after the first immunization.

#### Histological Evaluation

The morphological studies were performed with the imaging CellSens Dimention (Olympus) software. Osteoarthritis histopathology was assessed based on the symptoms associated with synovial inflammation, chondropathy, and subchondral osteoarthritis. Synovitis index estimation involved synovial inflammation, synovial lining thickness, dense of cellularity, and presence of perivascular lymphoid aggregates, where normal synovium (score 0) is defined as thin linning with sparse cellular distribution and no/few inflammatory cells, mild and moderate synovial inflammation (score 1 to 2) indicate thickness of linning with dense cellularity with inflammatory cells, and severe inflammation (score 3) means hypervascular synovitis containing lymphoid aggregates. Two aspects of chondropathy were graded: surface integrity and chondrocyte yielding score 0 for normal morphology to score 3 for chondral lesion with chondrocyte hypercellurarity and/or cloning, and complete cartilage surface integrity loss. Subchondral plate thickening, bone trabeculae deterioration, bone marrow lesion (bone pseudocysts - cavitary lesion formation) associated with fibrovascular replacement of marrow spaces were accounted for subchondral osteoarthritis with grading score from 0 to 3 in severe lesion. The sample preparation procedure for histological evaluation is described in the supplementary materials.

### Pharmacokinetic Studies

Pharmacokinetics of the studied compounds was investigated in female Lewis rats following IV and IP administration of GRMS-55 at doses of 20 and 40 mg kg^−1^, IV and SC administration of (±)-LSF at doses of 40 and 80 mg kg^−1^, and IP administration of rolipram at a dose of 10 mg kg^−1^. In addition, pharmacokinetics of GRMS-55 and rolipram was studied following IV administration to male Wistar rats. GRMS-55 was administered at a dose of 20 mg kg^−1^ and rolipram at a dose of 5 mg kg^−1^. The same vehicles as these in pharmacodynamic studies for each compound and route of administration were used. Rats were implanted with catheters into the jugular vein and after a two-day period they were administered with the investigated compounds by the listed above routes of administration. Blood samples were collected from the catheters to heparinized tubes at 5, 15, 30, 60, 90, and 120 min following (±)-LSF or GRMS-55 dosing and at 10, 20, 40, 80, 120, 160, and 220 min following rolipram administration to female Lewis rats. In turn, samples from male Wistar rats were collected at 5, 15, 30, 60, 90, and 120 min following GRMS-55 administration and 10, 20, 40, 80, and 120 min after rolipram dosing. Samples were subsequently centrifuged at 3000 × g for 10 min. Plasma was stored at −80°C until analysis.

### Analytical Methods

To analyze levels of (±)-LSF and its metabolite PTX in plasma of rats, the method described earlier was used (9). To determine GRMS-55 and rolipram concentrations in rat plasma, HPLC methods with UV detection were developed. A detailed description of these methods has been provided in the supplementary materials. The calibration curve of GRMS-55 was linear at the range of 0.050–20 and for rolipram at the range of 0.020–20 mg L^−1^.

TNF-α levels in LPS-stimulated rat blood *in vitro* and in rat plasma samples from the *in vivo* studies were analyzed by the Rat TNF-α Quantikine ELISA Immunoassay Kit (R&D Systems, USA) according to the manufacturer’s instructions. The assay was linear at the range of 12.5–800 pg mL^−1^. Levels of TNF-α in mice serum were measured by ELISA Kits for Tumor Necrosis Factor Alpha (Cloud-Clone Corp., USA). This assay was linear at the range of 15.6–1000 pg mL^−1^. Absorbance was measured at 450 nm using a POLARstar Omega multi-mode microplate reader (BMG LABTECH, Germany).

Serum asparagine transaminase (AST) and alanine transaminase (ALT) activity was analyzed using the chemistry analyzer BS-800 (Shenzhen Mindray Bio-medical Electronics Co. ltd, China).

### Pharmacokinetic, PK/PD, and PK/PD/DIS Progression Modeling

Firstly, the concentration-time data collected for each individual compound from one of the investigated rat strains (Wistar or Lewis) were subjected to a non-compartmental analysis (NCA). Subsequently, pharmacokinetic models were developed for each investigated compound and strain of rat. One- and two- compartment models with linear or non-linear elimination were tested. In the case of (±)-LSF administration, the existence of interconversion phenomenon between (±)-LSF and PTX, which was also observed in our previous studies (9), was taken into account during model development. The final models were chosen based on the goodness-of-fit criteria, such as Akaike Information Criterion (AIC), Schwarz Bayesian Criterion (SBC), weighted sum of squared residuals (WSSR), a visual inspection of fitting, and precision of estimated parameters expressed as coefficients of variation % (CVs%). NCA analysis was performed in Phoenix WinNonlin v. 7.0 (Pharsight Corp., a Certara Company, Princeton, New Jersey). Pharmacokinetic model parameters were estimated in ADAPT 5 software (BMSR, Los Angeles, CA) using the weighted least squares algorithm. Fraction of the dose absorbed (F) following an extravascular (EV) administration was calculated according to the Eq. 4:

4 $$ F=\frac{AUC_{0-t\left(\mathrm{EV}\right)}\cdotp {D}_{IV}}{AUC_{0-t\left(\mathrm{IV}\right)}\cdotp {\mathrm{D}}_{\mathrm{EV}}} $$

where *AUC*_*0-t*_ is the area under concentration *versus* time curve from 0 to the last measured point and D is dose.

PK/PD and PK/PD/DIS progression models were developed considering the mechanism of action of the investigated compounds, the mechanisms of disease progression in the case of CIA, and the subsequent stages leading to TNF-α release in the case of LPS-induced endotoxemia. Various numbers of transit compartments were tested during development of the models, and the final models were chosen based on the goodness-of-fit criteria, such as AIC, SBC, a visual inspection of the fitting, and parameter precision. Estimated values of pharmacokinetic parameters were fixed during PK/PD and PK/PD/DIS progression model fitting procedures. No influence of experimentally induced diseases (RA and LPS-induced endotoxemia) on the values of pharmacokinetic parameters of each investigated compound was found, as concentrations of the test compounds in blood collected from diseased rats were close to those predicted by the pharmacokinetic models developed in this study using healthy animals (data not shown). Pharmacokinetic parameters of (±)-LSF and PTX in male Wistar rats challenged with IV dose of 1 mg kg^−1^ of LPS were taken from our previous study (9). PK/PD and PK/PD/DIS progression modeling were performed in ADAPT 5 software using the weighted least squares algorithm.

#### PK/PD Model of PDE Inhibitors in Endotoxemic Rats

In the LPS-induced endotoxemia model, TNF-α concentrations were measured following IV administration of both (±)-LSF and its metabolite – PTX. As following administration of (±)-LSF or PTX to rats and other species both of these compounds occur simultaneously in plasma of an individual, the interaction PK/PD model was used to estimate pharmacodynamic parameters, including *IC*_*50*_ values, for each of these compounds. An additive interaction between (±)-LSF and PTX was assumed in the PK/PD model based on the results of the recent study, where this type of interaction was found between these compounds as modulators of cAMP levels (9). To study the influence of GRMS-55 and rolipram on TNF-α levels in this model, two other experiments with independent control groups were performed. Therefore, as a result of PK/PD analysis, three sets of pharmacodynamic parameters were obtained based on the data from the three individual experiments.

**Fig. 1:**
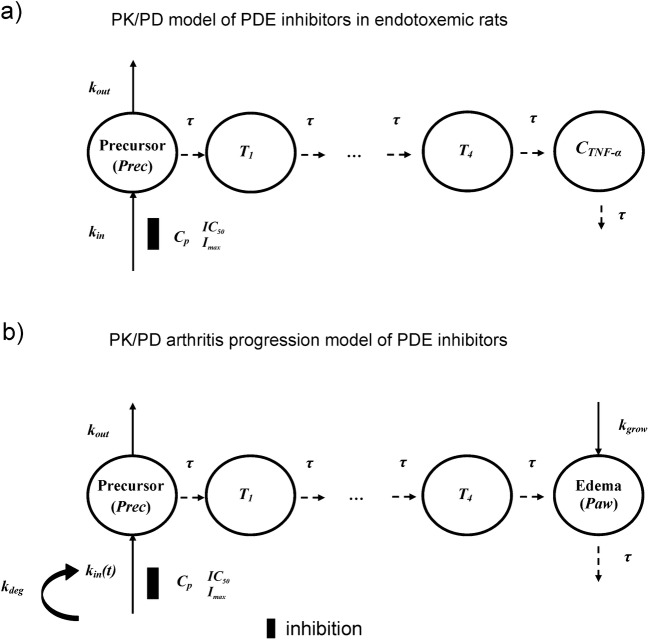
Schematic representation of the proposed PK/PD model to describe changes in TNF-α plasma levels in male Wistar rats with LPS-induced endotoxemia **a** and PK/PD/DIS model of arthritis progression in female Lewis rats **b** treated with the studied compounds; *Prec* are the hypothetical precursors of TNF-α production **a** or relative paw edema **b** in the precursor compartments, *I*_*max*_ is the maximal inhibitory effect of each compound, *C*_*p*_ is a plasma concentration of the test compound (GRMS-55, (±)-LSF, rolipram, and additionally PTX in the case of endotoxemic rats), *IC*_*50*_ is the concentration of each compound that produce 50% of *I*_*max*_, *Paw* is a relative paw swelling, *τ* is the mean transit time, *k*_*in*_ is the zero-order rate constant of production of *Prec*, while *k*_*in*_*(t)* is the zero-order production component of *Prec, k*_*deg*_ is the first-order rate constant of *k*_*in*_*(t)* reduction, T_1_-T_4_ are precursors of TNF-α production **a** or relative paw edema **b** in subsequent transit compartments, k_out_ is the first-order rate constant of *Prec* degradation, and k_grow_ is the zero-order rate constant accounting for natural paw growth.

A structure of the developed PK/PD model for TNF-α inhibitory effect of studied compounds is illustrated in Fig. 1a.

The applied model is a modified indirect response model (19) with implemented transit compartments (*T*_*1*_-*T*_*4*_) that describe the delay of the observed pharmacodynamic response. In this scheme *τ* is the mean transit time, *k*_*in*_ is the time limited (from 0 to *t*_*lag*_) zero-order rate constant of production of a TNF-α precursor (e.g., TNF-α mRNA). Different numbers of transit compartments were tested during the model development, but the best fit was observed using four transit compartments. Pharmacokinetic parameters of GRMS-55 (Eq. 5) and rolipram (Eqs. 6 and 7) in male Wistar rats were estimated prior to the PK/PD analysis by solving the following differential equations:

5 $$ {V}_{GRMS-55}\cdotp \frac{d{C}_{GRMS-55}}{dt}=-{CL}_{t\left( GRMS-55\right)}\cdotp {C}_{GRMS-55} $$

6 $$ {\displaystyle \begin{array}{c}{V}_{1(ROL)}\cdotp \frac{d{C}_{1(ROL)}}{dt}=-\left({CL}_{t(ROL)}+{CL}_{d(ROL)}\right)\cdotp {C}_{1(ROL)}+{CL}_{d(ROL)}\cdotp {C}_{2(ROL)}\\ {}\kern5.5em \end{array}} $$

7 $$ {V}_{2(ROL)}\cdotp \frac{d{C}_{2(ROL)}}{dt}={CL}_{d(ROL)}\cdotp \left({C}_{1(ROL)}-{C}_{2(ROL)}\right) $$

where *C*_*GRMS-55*_ and *C*_*1(ROL)*_ are concentrations of each compound in the central compartments, while *C*_*2(ROL)*_ is rolipram concentration in the peripheral compartment. *Cl*_*t(GRMS-55)*_ and *Cl*_*t(ROL)*_ are the total clearances, and *V*_*GRMS-55*_ and *V*_*1(ROL)*_ are volumes of central compartments for each compound, while *V*_*2(ROL)*_ is the volume of peripheral compartment for rolipram. *Cl*_*d(ROL)*_ is the distribution clearance of rolipram.

Pharmacodynamic parameters of the proposed PK/PD model were obtained by solving the following differential equations:

8 $$ \frac{dPrec}{dt}=\left\{\begin{array}{c}{k}_{in}\cdotp \left(1-\frac{I_{maxPTX}\cdotp {C}_{PTX}}{IC_{50\mathrm{PTX}}+{C}_{PTX}}\right)\cdotp \left(1-\frac{I_{maxLSF}\cdotp {C}_{LSF}}{IC_{50\mathrm{LSF}}+{C}_{LSF}}\right)-{k}_{out}\cdotp Prec,\kern0.75em t<{t}_{lag},\kern0.5em Prec(0)={R}_0\\ {}0,\kern26em t\ge {t}_{lag}\kern7.5em \end{array}\right. $$

9 $$ \frac{dPrec}{dt}=\left\{\begin{array}{c}{k}_{in}\cdotp \left(1-\frac{I_{max}\cdotp {C}_p}{IC_{50}+{C}_p}\right)-{k}_{out}\cdotp Prec,\kern11.5em t<{t}_{lag},\kern0.5em Prec(0)={R}_0\\ {}0,\kern24em t\ge {t}_{lag}\kern7.5em \end{array}\right. $$

10 $$ \frac{d{T}_1}{dt}=\left( Prec-{T}_1\right)\cdotp \frac{1}{\tau },\kern6.5em {T}_1(0)={R}_0 $$

11 $$ \frac{d{T}_{n+1}}{dt}=\left({T}_n-{T}_{n+1}\right)\cdotp \frac{1}{\tau },\kern5.9em {T}_{n+1}(0)={R}_0,\kern0.75em n=1\sim 3 $$

12 $$ \frac{d{C}_{TNF}}{dt}=\left({T}_4-{C}_{TNF}\right)\cdotp \frac{1}{\tau },\kern4.90em {C}_{TNF}(0)={R}_0 $$

The Eq. 8 was used when fitting the model to the data from the experiment on PTX and (±)-LSF, while Eq. 9 in the case of GRMS-55 or rolipram. *I*_*max*_*, I*_*maxPTX*_*,* and *I*_*maxLSF*_ are the maximal inhibitory effects of each compound on the production rate constant (*k*_*in*_), *C*_*p*_, *C*_*PTX*_, and *C*_*LSF*_ are plasma concentrations of the compounds, and *IC*_*50*_, *IC*_*50PTX*_ and *IC*_*50LSF*_ are concentrations of each compound that produce 50% of *I*_*max*_, *Prec* is a concentration of precursor in the precursor compartment, *T*_*n*_ is a concentration of transmitter in the *n*^*th*^ transit compartment, *C*_*TNF*_ is a concentration of TNF-α in rat plasma, *R*_*0*_ is the basal plasma concentration of TNF-α in healthy rats equal to 12.7 pg mL^−1^, calculated as the average of concentrations measured in samples collected from 4 healthy male Wistar rats. All *I*_*max*_ values were fixed to 1.

#### PK/PD/Arthritis Progression Modeling of PDE Inhibitors

**Fig. 2:**
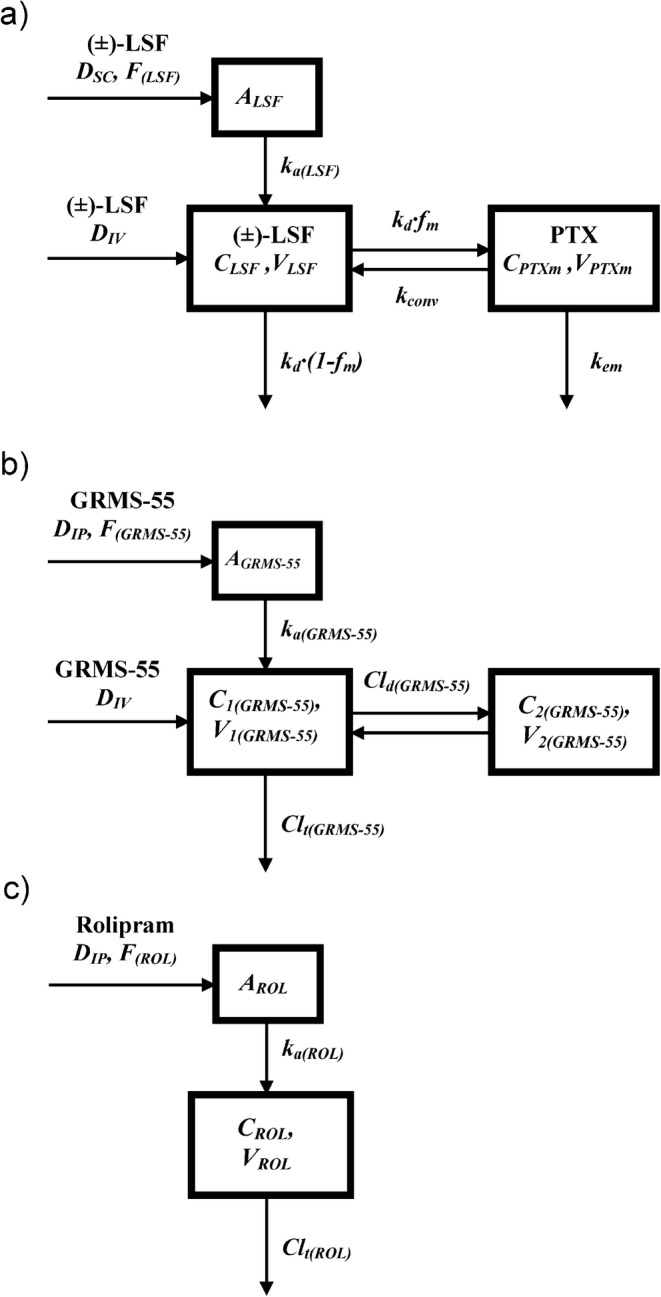
Schematic representation of the pharmacokinetic models of (±)-LSF **a**, GRMS-55 **b**, and rolipram **c** following IV and SC administration of (±)-LSF at doses of 40 and 80 mg kg-^1^, IV and IP administration of GRMS-55 at doses of 20 and 40 mg kg-^1^, respectively, and IP administration of rolipram at a dose of 10 mg kg^−1^ to female Lewis rats; *A*_*GRMS-55*_, *A*_*LSF*_, and *A*_*ROL*_ are amounts of each compound in the absorption compartments; *F*_*(GRMS-55)*_, *F*_*(LSF)*,_ and *F*_*(ROL)*_ are the fractions absorbed following EV administration of GRMS-55, (±)-LSF, and rolipram, respectively; *k*_*a(ROL)*_, *k*_*a(GRMS-55)*_, and *k*_*a(LSF)*_ are the first-order absorption rate constants of each compound following EV dosing; *C*_*1(GRMS-55)*_, *C*_*LSF*_, *C*_*ROL*_, and *C*_*PTXm*_ are plasma concentrations of GRMS-55, (±)-LSF, rolipram, and PTX as a metabolite, while *C*_*2(GRMS-55)*_ is the concentration of GRMS-55 in the peripheral compartment; *V*_*1(GRMS-55)*_, *V*_*LSF*_, *V*_*ROL*_, and *V*_*PTXm*_ are volumes of central compartments for each compound, while *V*_*2(GRMS-55)*_ is the volume of peripheral compartment for GRMS-55; *Cl*_*d(GRMS-55)*_ is the distribution clearance of GRMS-55; *k*_*d*_ is the first-order rate constant for disappearance of (±)-LSF; *f*_*m*_ is a fraction of (±)-LSF metabolized; *k*_*conv*_ is the first-order rate constant of conversion of PTX to (±)-LSF; *Cl*_*t(GRMS-55)*_ and *Cl*_*t(ROL)*_ are the total clearances, *k*_*em*_ is the first-order rate constant of PTX elimination.

In the pharmacokinetic study, the female Lewis rats were administered with (±)-LSF by IV and SC routes, GRMS-55 by IV and IP routes, and rolipram by IP route. The route of (±)-LSF administration was selected based on the results of our recent study, where SC route of administration was recognized as the most preferential for this compound (20). The concentration-time data for each of the investigated compounds were simultaneously fitted to obtain a single set of pharmacokinetic parameters. Schematic representations of the applied pharmacokinetic models are presented in Fig. 2.

Pharmacokinetic parameters of GRMS-55 (Eqs. 13, 14, and 15), rolipram (Eqs. 16 and 17), and LSF (Eqs. 18, 19, and 20) in female Lewis rats have been estimated prior to the PK/PD analysis by solving the following differential equations:

13 $$ {V}_{1\left( GRMS-55\right)}\cdotp \frac{d{C}_{1\left( GRMS-55\right)}}{dt}=-\left({CL}_{t\left( GRMS-55\right)}+{CL}_{d\left( GRMS-55\right)}\right)\cdotp {C}_{1\left( GRMS-55\right)}+{k}_{a\left( GRMS-55\right)}\cdotp {A}_{GRMS-55}+{CL}_{d\left( GRMS-55\right)}\cdotp {C}_{2\left( GRMS-55\right)} $$

14 $$ \frac{d{A}_{GRMS-55}}{dt}=-{k}_{a\left( GRMS-55\right)}\cdotp {A}_{GRMS-55} $$

15 $$ {V}_{2\left( GRMS-55\right)}\cdotp \frac{d{C}_{2\left( GRMS-55\right)}}{dt}={CL}_{d\left( GRMS-55\right)}\cdotp \left({C}_{1\left( GRMS-55\right)}-{C}_{2\left( GRMS-55\right)}\right) $$

16 $$ {\displaystyle \begin{array}{c}{V}_{ROL}\cdotp \frac{d{C}_{ROL}}{dt}=-{CL}_{t(ROL)}\cdotp {C}_{ROL}+{k}_{a(ROL)}\cdotp {A}_{ROL}\\ {}\kern3em \end{array}} $$

17 $$ \frac{d{A}_{ROL}}{dt}=-{k}_{a(ROL)}\cdotp {A}_{ROL} $$

18 $$ \frac{d{X}_{LSF}}{dt}=-{k}_d\cdotp {X}_{LSF}+{k}_{a(LSF)}\cdotp {A}_{LSF}+{k}_{conv}\cdotp {X}_{PTXm} $$

19 $$ \frac{d{X}_{PTXm}}{dt}=-{k}_{em}\cdotp {X}_{PTXm}+{k}_d\cdotp {X}_{LSF}-{k}_{conv}\cdotp {X}_{PTXm} $$

20 $$ \frac{d{A}_{LSF}}{dt}=-{k}_{a(LSF)}\cdotp {A}_{LSF} $$

where *X*_*LSF*_ is an amount of (±)-LSF in the central compartment and *X*_*PTXm*_ is an amount of PTX in the metabolite compartment divided by the fraction metabolized (*f*_*m*_). Concentrations of (±)-LSF and PTX in these compartments are equal to *X*_*LSF*_*/V*_*LSF*_ and *X*_*PTXm*_· *f*_*m*_*/V*_*PTXm*_*,* respectively. The remaining symbols and parameters have been explained and defined in Fig. 2 and Table II.

To describe changes in paw size in rats with CIA, several PK/PD/DIS progression models described in the literature were tested, including the modified indirect response model developed by Earp *et al.* (18) and the transduction-based feedback model (21), but the model developed in this study demonstrated the best fitting to the experimental data collected. In this model, the effect of PDE inhibitors was described by the type I indirect response model with signal transduction, which incorporates an inhibitory effect on the production of a precursor. A precursor is not defined, but it may be a certain cytokine, such as TNF-α or its mRNA, as PDE inhibitors are recognized inhibitors of cytokine synthesis and release. A schematic representation of the proposed PK/PD/DIS progression model of CIA is illustrated in Fig. 1b. From this figure, the developed model is a modified type I indirect response model with the implemented production component represented by the rate *k*_*in*_*(t)*, which is a function of time that is reduced by the loss of production first-order rate constant (*k*_*deg*_), representing a linear decline in the variable *k*_*in*_*(t)* and accounts for the natural remission of arthritis after disease onset. *k*_*grow*_ is the zero-order rate constant of a natural paw growth, *τ* is the mean transit time, *k*_*out*_ is the first-order disappearance rate constant of the precursor of paw edema. Transit compartments (*T*_*1*_-*T*_*4*_) have been used in the model to describe a signal transduction leading to paw swelling.

The values of pharmacodynamic parameters of PK/PD/DIS model of arthritis progression were estimated by solving the following differential equations:

21 $$ \frac{dPrec}{dt}=\left\{\begin{array}{c}0,\kern15.5em t<{t}_{onset},\kern0.75em Prec(0)=1\\ {}{k}_{in}(t)\cdotp \left(1-\frac{I_{max}\cdotp {C}_p}{IC_{50}+{C}_p}\right)-{k}_{out}\cdotp Prec,\kern2.25em t\ge {t}_{onset}\kern7em \end{array}\right. $$

22 $$ \frac{d{k}_{in}}{dt}=-{k}_{deg}\cdotp {k}_{in},\kern10.5em {k}_{in}(0)={k}_{in}^0\kern2em $$

23 $$ \frac{d{T}_1}{dt}=\left( Prec-{T}_1\right)\cdotp \frac{1}{\tau },\kern8em {T}_1(0)=1 $$

24 $$ \frac{d{T}_{n+1}}{dt}=\left({T}_n-{T}_{n+1}\right)\cdotp \frac{1}{\tau },\kern6.9em {T}_{n+1}(0)=1,n=1\sim 3 $$

25 $$ \frac{dPaw}{dt}=\left({T}_4- Paw\right)\cdotp \frac{1}{\tau }+{k}_{grow},\kern2.5em Paw(0)=1 $$

*Prec* is a hypothetical precursor of relative paw edema in the precursor compartment, *t*_*onset*_ is the delay in production of the precursor accounting for the delay in disease onset, and *k*_*in(0)*_ is the initial condition of *k*_*in*_*(t)* variable.

### Statistical Analysis

All statistical calculations were performed in IBM SPSS Statistics 24 (IBM Corp., USA) or STATISTICA 13 (Tibco Software Inc., USA). Analyses of parametric data were performed using the Student’s t test or a one-way analysis of variance (ANOVA) followed by an appropriate post-hoc test in the case of more than 2 groups tested. Non-parametric data were compared using Mann-Whitney U or Kruskal–Wallis tests in the case of more than 2 groups tested. *P* values less than 0.05 were considered as statistically significant.

## Results

### PDE Assay

Inhibitory activities of the investigated and reference compounds against various hrPDEs were examined and the values of *IC*_*50*_, *I*_*max*_, and *γ* parameters of the studied inhibitors have been listed in Table 1.

**Table 1:** Inhibitory Activities of the Studied Compounds Against Different hrPDEs, Expressed as Estimated Values of *IC*_*50*_, *I*_*max*_*,* and ***γ*** Parameters

Enzyme Comp.		PDE1B	PDE2A	PDE3A	PDE4B	PDE4D	PDE5A	PDE7A	PDE10A
		Estimate (CV%)							
GRMS-55	*IC*_*50*_ [μM] *I*_*max*_ *γ*	2.5 (22.7) 93 (1.8) 1.0 (19.1)	>100	13.4 (8.6) 98 (3.2) 1.5 (10.3)	26.9^a^ (27.8) 100 (10.7) 1.2 (34.5)	>200	>100	7.3^a^ (24.6) 96 (5.3) 0.97 (24.1)	23.3 (5.1) 96 (2.7) 3.0 (13.6)
(±)-LSF	*IC*_*50*_ [μM] *I*_*max*_ *γ*	12.9 (15.5) 97 (3.6) 1.0 (17.8)	91.6 (12.1) 83 (9.1) 2.1 (17.0)	73.4 (5.18) 99 (3.5) 2.5 (12.6)	50.8 (3.6) 98 (4.0) 2.5 (23.7)	>200	>100	297.2 (10.5) 100 (4.6) 2.5 (25.7)	>100
Vinpocetine	*IC*_*50*_ [μM] *I*_*max*_ *γ*	7.5 (15.0) 99 (1.1) 1.5 (17.1)							
EHNA	*IC*_*50*_ [μM] *I*_*max*_ *γ*		2.8 (8.7) 79 (2.4) 1.4 (9.9)						
Milrinone	*IC*_*50*_ [μM] *I*_*max*_ *γ*			0.2 (8.4) 99 (0.4) 1.4 (7.9)					
Rolipram	*IC*_*50*_ [μM] *I*_*max*_ *γ*				1.1^a^ (7.1) 99 (0.3) 1.6 (7.7)	5.0 (17.4) 99 (5.6) 1.7 (25.5)			
Zaprinast	*IC*_*50*_ [μM] *I*_*max*_ *γ*						5.1 (33.5) 99 (7.1) 0.7 (21.2)		
BRL-50481	*IC*_*50*_ [μM] *I*_*max*_ *γ*							2.1^a^ (7.3) 98 (2.4) 2.5 (15.6)	
Papaverine	*IC*_*50*_ [μM] *I*_*max*_ *γ*								0.066 (4.5) 99 (0.9) 1.6 (6.7)

^a^data already published (9,16)

From this table, GRMS-55 exhibited a strong inhibitory activity (comparable to that of reference compounds) against hrPDE1B and hrPDE7A. In addition, a moderate activity of this compound on hrPDE3A and hrPDE4B and a slight activity against hrPDE10A were observed. GRMS-55 did not inhibit hrPDE2A, hrPDE4D, and hrPDE5A at the range of concentrations tested. On the other hand, (±)-LSF displayed a moderate inhibitory activity against hrPDE1B and hrPDE4B, and only a slight activity was noted in the cases of hrPDE2A and hrPDE3A. The reference compounds, being selective or preferential inhibitors of individual isoenzymes, exhibited micromolar activity, while papaverine and milrinone demonstrated *IC*_*50*_ values below 1 μM on hrPDE10A and hrPDE3A, respectively. Both GRMS-55 and (±)-LSF demonstrated an inhibitory activity against hrPDE4B, which is crucial for achieving the anti-inflammatory and immunomodulatory effects (1). However, they were considerably weaker than the reference compound – rolipram, i.e., 24 times in the case of GRMS-55 and 45 times for (±)-LSF. Nevertheless, none of them demonstrated activity against hrPDE4D at a range of concentrations tested, inhibition of which is supposed to be associated with the occurrence of nausea and vomiting. In contrast, rolipram exhibited a strong inhibitory activity on hrPDE4D. The abovementioned undesired symptoms are frequently observed side effects following administration of PDE4 inhibitors (15). The observed selectivity of the investigated compounds in relation to PDE4B *vs.* PDE4D may be essential for their suitability for therapeutic applications. The representative inhibition graphs illustrating inhibitory properties and fittings of the pharmacodynamic model to the data of each investigated and reference compound have been placed in the supplementary materials (Fig. 1S).

### TNF-α Inhibition ***In Vitro***

**Fig. 3:**
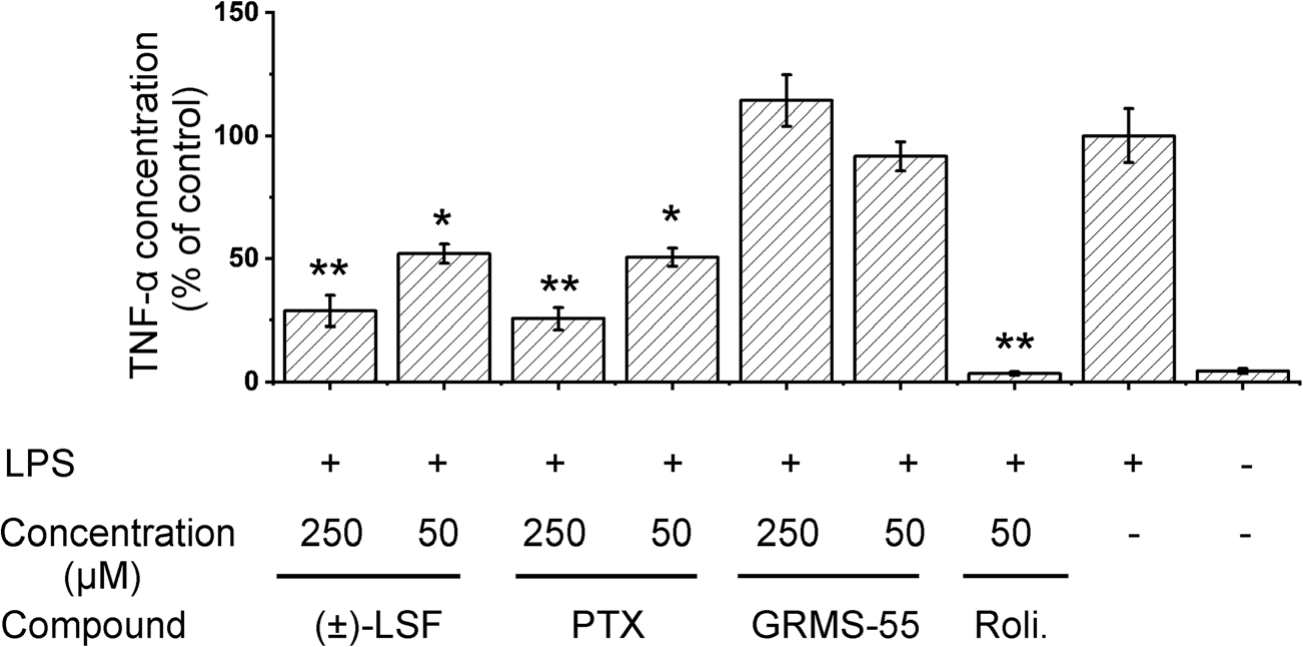
Inhibition of LPS-induced TNF-α release in whole rat blood by the investigated and reference compounds. Each value represents the mean (±SD) % of the control (vehicle + LPS) of four independent cultures. ** *p* < 0.001, * *p* < 0.05 compared to the control group (vehicle + LPS), using Tamhane’s T2 multiple comparison parametric test for unequal variance.

The results of *in vitro* study of TNF-α release inhibition in whole rat blood stimulated with LPS have been illustrated in Fig. 3.

The graph shows that both PTX and (±)-LSF significantly and with similar potency reduced levels of TNF-α, while GRMS-55 did not exhibit activity in this test compared to the LPS-treated control. Rolipram, a potent PDE4 inhibitor, at a concentration of 50 μM completely reduced the levels of TNF-α in LPS-stimulated rat blood compared to the control group. Moreover, the activity of this compound was significantly higher than those of PTX and (±)-LSF. It is worth noting that (±)-LSF and PTX inhibited TNF-α release in a dose-dependent manner.

### Pharmacokinetics

(±)-LSF demonstrated a linear pharmacokinetics in female Lewis rats, as a 1-compartment model with a metabolite compartment and linear elimination well described the concentration *vs.* time profiles of this compound and PTX as a metabolite. GRMS-55 displayed a biphasic, linear pharmacokinetics corresponding to a 2-compartment model, while the concentration-time data obtained after IP administration of rolipram were best described by a 1-compartment model. In the pharmacokinetic experiments performed in male Wistar rats, both GRMS-55 and rolipram were administered by IV route. The concentration-time data from the study on GRMS-55 were best captured by a 1-compartment pharmacokinetic model, while rolipram displayed a biphasic pharmacokinetics in male Wistar rats. Pharmacokinetic models employed in this study were simultaneously fitted to the data to obtain a single set of pharmacokinetic parameters for each investigated compound and rat strain.

**Fig. 4:**
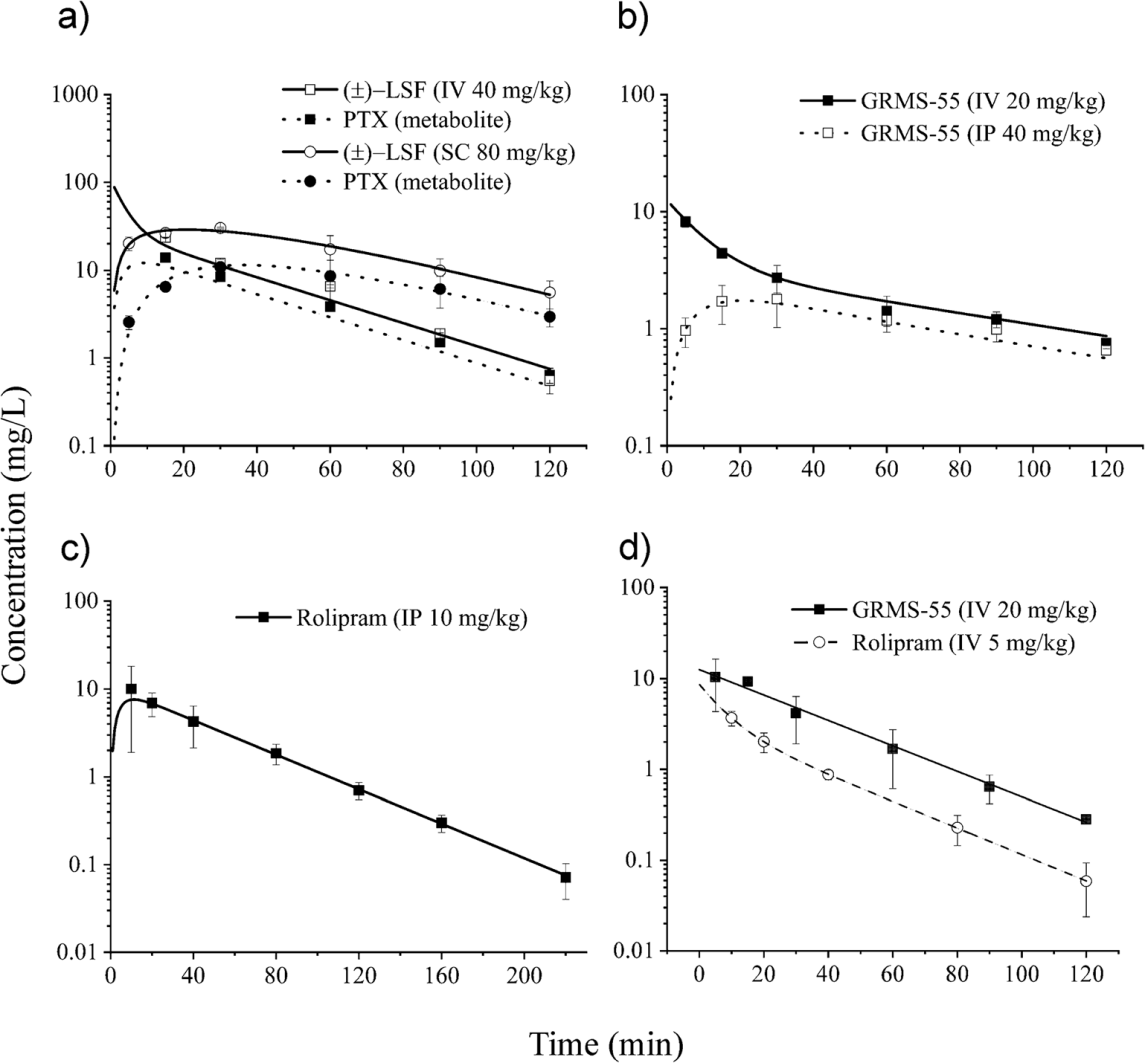
Mean (±SD) observed (symbols) and predicted by the models (lines) (±)-LSF and PTX, GRMS-55, and rolipram plasma concentrations following (±)-LSF **a**, GRMS-55 **b**, or rolipram **c** administration to female Lewis rats and IV administration of rolipram or GRMS-55 to male Wistar rats (d) (*n* = 3–4).

As illustrated in the graphs (Fig. 4a-c), the model predicted concentration *vs.* time profiles of the investigated compounds in female Lewist rats fairly well captured their measured levels in rat plasma. The estimated by the models values of pharmacokinetic parameters are collected in Table II.

**Table II:** Values of Pharmacokinetic Parameters of (±)-LSF, GRMS-55, Rolipram, and PTX as a Metabolite in Female Lewis and Male Wistar Rats

Rolipram			GRMS-55			(±)-LSF		
Parameter	Brief description	Estimate (CV%)	Parameter	Brief description	Estimate (CV%)	Parameter	Brief description	Estimate (CV%)
Female Lewis rats								
*V*_*(ROL)*_*/F* (L kg^-1^)	Volume of distribution divided by fraction absorbed	1.02 (2.9)	*V*_*1(GRMS-55)*_ (L kg^-1^)	Volume of distribution	1.60 (15.4)	*V*_*LSF*_ (L kg^-1^)	Volume of distribution of (±)-LSF	0.37 (12.5)
*k*_*a(ROL)*_ (min^-1^)	Absorption rate constant	0.228 (15.4)	*k*_*a(GRMS-55)*_ (min^-1^)	Absorption rate constant	0.045 (17.6)	*V*_*PTXm*_*/f*_*m*_ (L kg^-1^)	Volume of distribution divided by fraction metabolized	1.95 (21.2)
*Cl*_*t*_*/F*_*(ROL)*_ (L min^-1^)	Total clearance divided by fraction absorbed	0.023 (2.1)	*Cl*_*t(GRMS-55)*_ (L min^-1^)	Total clearance	0.053 (10.8)	*k*_*a(LSF)*_ (min^-1^)	Absorption rate constant	0.032 (13.8)
			*Cl*_*d(GRMS-55)*_ (L min^-1^)	Distribution clearance	0.080 (28.6)	*k*_*d*_ (min^-1^)	Overall parent elimination rate constant	0.212 (27.1)
			*V*_*2(GRMS-55)*_ (L kg^-1^)	Volume of peripheral compartment	2.14 (29.6)	*k*_*em*_ (min^-1^)	Metabolite elimination rate constant	0.039 (12.1)
						*k*_*conv*_ (min^-1^)	Conversion rate constant of PTX to (±)-LSF	0.054 (29.1)
Male Wistar rats								
*V*_*1(ROL)*_ (L kg^-1^)	Volume of distribution	0.58 (11.6)	*V*_*(GRMS-55)*_ (L kg^-1^)	Volume of distribution	1.59 (9.2)	*V*_*LSF*_ (L kg^−1^)	Volume of distribution of (±)-LSF	1.22 (3.4)^a^
*Cl*_*t(ROL)*_ (L min^-1^)	Total clearance	0.037 (2.6)	*Cl*_*t(GRMS-55)*_ (L min^-1^)	Total clearance	0.051 (6.4)	*V*_*PTXm*_ (L kg^−1^)	Volume of distribution of PTX	2.01 (9.5)^a^
*Cl*_*d(ROL)*_ (L min^-1^)	Distribution clearance	0.021 (16.0)				*V*_*max*_ (mg min^−1^ kg^−1^)	Maximal elimination rate constant of (±)-LSF	0.433 (56.9)^a^
*V*_*2(ROL)*_ (L kg^-1^)	Volume of peripheral compartment	0.28 (10.0)				*K*_*m*_ (mg L^−1^)	Michaelis-Menten constant	6.02 (91.8)^a^
						*k*_*m12*_ (min^−1^)	Distribution rate constant of PTX	0.203 (12.7)^a^
						*k*_*m21*_ (min^−1^)	Redistribution rate constant of PTX	0.095 (12.9)^a^
						*k*_*em*_ (min^−1^)	Elimination rate constant of PTX	0.116 (9.8)^a^

^a^data already published (10)

A flip-flop pharmacokinetics was observed following SC administration of (±)-LSF. Thus, despite the high value of *k*_*d*_ constant, the rate of (±)-LSF elimination was limited by 6.6 times lower rate of its absorption, and therefore, the plasma terminal half-life was equal to 23 min. The values of GRMS-55 and rolipram elimination rate constants estimated by the models were comparable and, in consequence, half-life values calculated as *ln2·V*_*1*_*/Cl*_*t*_ were similar and equal to 21 and 30 min, respectively. Calculated from the Eqs. 4 fraction of the dose absorbed was 23% following IP administration of GRMS-55 and 95% following SC administration of (±)-LSF. In the case of (±)-LSF this value was similar to that observed in male Wistar rats, confirming the earlier observation that SC route is the most preferential EV route of administration of this compound (20). The volume of distribution of GRMS-55 was considerably larger (~2.7 times) and the elimination rate constant of this compound was approx. 2 times lower compared to those of rolipram. In contrast to the experiment in female Lewis rats, the concentration-time data obtained following the GRMS-55 dosing to male Wistar rats were better described by a 1-compartment pharmacokinetic model, while a 2-compartment model was more suitable to capture the pharmacokinetic profile of rolipram (Fig. 4d). Pharmacokinetics of (±)-LSF and PTX in male Wistar rats has already been investigated in our recent study (9), so the values of pharmacokinetic parameters of (±)-LSF and PTX estimated in that study (Table 2) were used in further PK/PD analysis of these compounds in the LPS-induced endotoxemia model.

### ConA-Induced Hepatitis

**Fig. 5:**
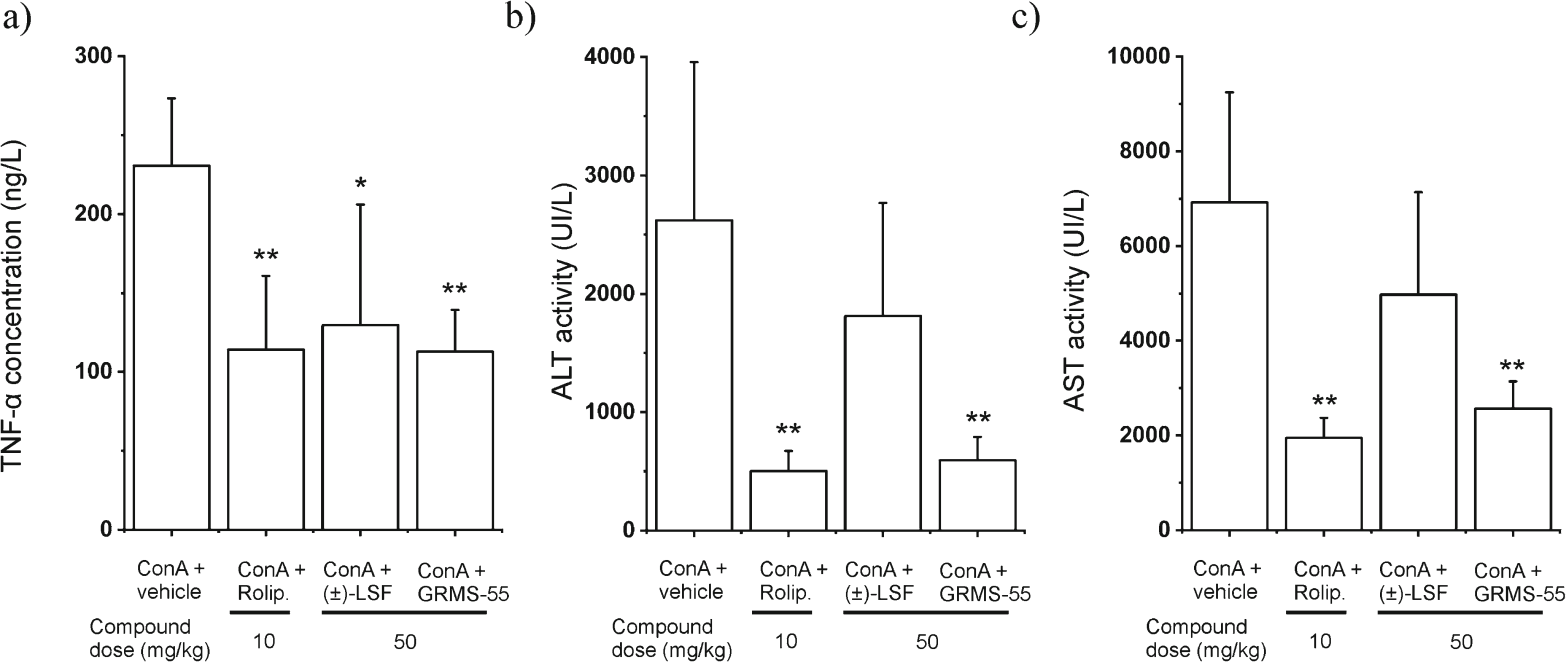
Inhibition of TNF-α secretion (a) and a decrease in ALT (b) and AST (c) activities in ConA-induced hepatitis in mice by the investigated and reference compounds 8 h following ConA administration. Each value represents the mean (+SD) level of each biomarker in mice serum (*n* = 6). * p < 0.05, ** *p* < 0.01, a one-way ANOVA with Tukey HSD post-hoc test.

Figure 5 shows the results of experiment performed in BALB/c mice, which were pre-treated with the investigated and reference PDE inhibitors or vehicle alone (control group) and subsequently administered with IV dose of ConA.

The data presented in the bar charts indicate that rolipram at a dose of 10 mg kg^−1^ as well as GRMS-55 at a dose of 50 mg kg^−1^ most potently inhibited TNF-α release (*p* < 0.01) and significantly reduced (*p* < 0.01) the activities of transaminases in mice sera compared to the control group 8 h following ConA administration. (±)-LSF, which is the weakest inhibitor of PDE4B and PDE7A out of the investigated compounds, demonstrated only a moderate activity at a dose of 50 mg kg^−1^ significantly reducing TNF-α levels, however, it failed to decrease the activities of transaminases. These results indicate that selective inhibition of PDE4 is responsible for the observed anti-inflammatory and hepatoprotective effects in ConA-induced hepatitis. Moreover, the PDE4B inhibition seems to be mainly engaged in these activities and a blockage of PDE4D is not required to achieve these effects.

### LPS-Induced Endotoxemia

**Fig. 6:**
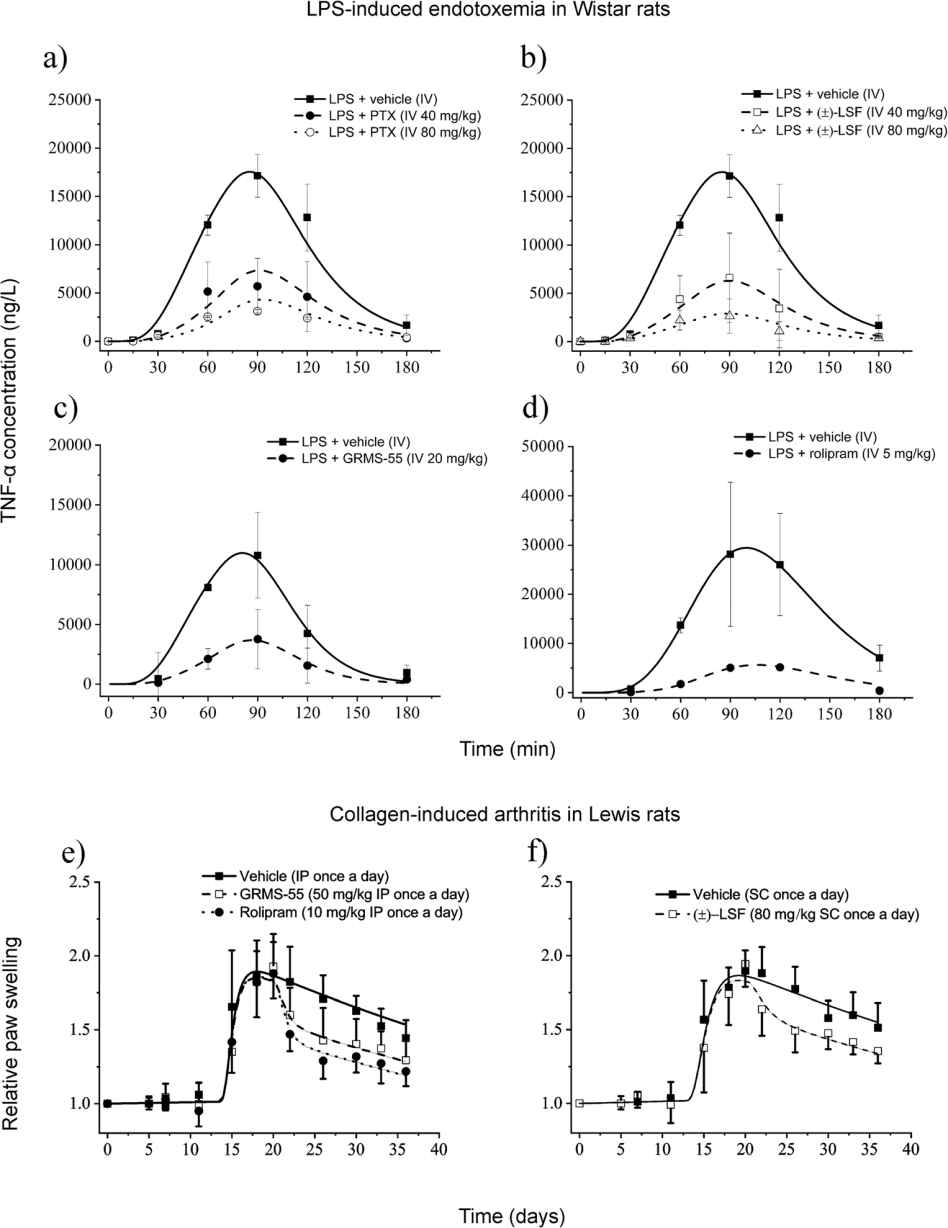
Mean (±SD) observed (symbols) and predicted (lines) based on the PK/PD model developed in this study plasma TNF-α concentrations following a single IV dose of (±)-LSF or PTX **a** and **b**, GRMS-55 **c**, or rolipram **d** to male Wistar rats with LPS-induced endotoxemia (*n* = 4); Time course of mean (±SD) observed (symbols) and predicted (lines) based on the PK/PD/DIS progression model developed in this study relative paw swelling following multiple administration of rolipram or GRMS-55 **e** and (±)-LSF **f** to female Lewis rats with CIA (n = 6).

Figure 6a-d shows the mean observed and predicted by the PK/PD model concentrations of TNF-α in plasma of rats challenged with IV dose of LPS in the presence and absence of the studied compounds.

From these graphs, TNF-α changes in rat plasma were satisfactorily predicted by the proposed PK/PD model. Pharmacodynamic parameters for each of the investigated compounds are summarized in Table III.

**Table III:** Pharmacodynamic Parameters Estimated Using the PK/PD Model Presented in Fig. 1a Following IV Administration of GRMS-55, (±)-LSF, PTX, or Rolipram to Rats Challenged with LPS

		GRMS-55	Rolipram	(±)-LSF	PTX
Parameter	Brief description	Final estimate (CV%)	Final estimate (CV%)	Final estimate (CV%)	
*k*_*out*_ (min^−1^)	Degradation of TNF-α precursor rate constant	0.059 (41.5)	0.031 (10.2)	0.034 (15.9)	
*k*_*in*_ (ng L^−1^ min^−1^)	Production of TNF-α precursor rate constant	0.809 (18.5)	1.83 (23.31)	0.827 (16.16)	
*t*_*lag*_ (min)	Time of the precursor production	57.1 (19.5)	48.6 (15.1)	60.0 (13.4)	
*τ* (min)	Mean transit time	8.0 (27.7)	12.0 (10.2)	7.3 (15.1)	
*IC*_*50*_ (mg L^−1^)	Concentration of compound producing 50% of I_max_	1.06 (77.3)	0.36 (18.9)	5.80 (19.7)	14.00 (32.2)
*I*_*max*_	Maximal inhibitory activity	1^a^	1^a^	1^a^	1^a^

^a^fixed during fitting procedure

From this table, the strongest activity was observed in the case of rolipram, and it was ~3, ~16, and ~39 times higher than those of GRMS-55, (±)-LSF, and PTX. PTX was considerably weaker than (±)-LSF in inhibiting TNF-α release *in vivo* as indicated by the relatively high *IC*_*50*_ value of PTX estimated by the model. Thus, considering that rather low levels of PTX are attained in plasma of rats administered with (±)-LSF, it can be concluded that the influence of PTX on TNF-α release after administration of (±)-LSF is minor.

### Collagen-Induced Arthritis

This study was performed in two independent experiments due to the fact that two different routes of administration of compounds and vehicles in each experiment were used. Model fittings of the relative paw swelling data from the three treatment groups and two control groups are presented in Fig. 6e and f. From these graphs, the applied PK/PD/DIS model well described the changes of relative paw edema in all investigated groups. There was observed a slight increase in paw size during the first 13 days of the experiment corresponding to a natural growth of a paw, and then a rapid increase in a paw edema with the peak at ~20th day post the first immunization. Subsequently, paw size began to decrease gradually in all investigated groups. However, in the groups treated with the investigated compounds the initial reduction in paw edema was more rapid. At the last observation time point, the relative paw swelling decreased by 35.2, 31.5, and 30.5% after rolipram, GRMS-55, and (±)-LSF administration, respectively. The values of pharmacodynamic and disease progression parameters are listed in Table IV.

**Table IV:** Parameters Estimated Using the PK/PD/DIS Arthritis Progression Model Presented in Fig. 1b Following Multiple Administration of GRMS-55, (±)-LSF, or Rolipram to Rats with Experimentally Induced Arthritis

		(±)-LSF	GRMS-55	Rolipram
Parameter	Brief description	Final estimate (CV%)	Final estimate (CV%)	
*k*_*out*_ (day^-1^)	Degradation of precursor rate constant	0.258 (1.7)	0.281 (1.6)	
*k*_*deg*_ (day^-1^)	Loss of production rate constant	0.0133 (13.4)	0.0127 (15.4)	
*t*_*onset*_ (day)	Time of disease onset	12.6 (3.9)	13.3 (2.3)	
*τ* (day)	Mean transit time	0.371 (19.7)	0.257 (16.8)	
*IC*_*50*_ (mg L^-1^)	Concentration producing 50% of maximal inhibition	1.06 (77.3)	0.26 (45.0)	0.01 (75.5)
*k*_*grow*_ (day^-1^)	Natural growth rate constant	0.004^a^	0.004^a^	0.004^a^
*k*_*in(0)*_ (day^-1^)	Initial value of precursor production variable	0.498 (1.1)	0.521 (1.5)	0.523 (1.2)
*I*_*max*_	Maximal inhibitory potency	1^b^	1^b^	1^b^

^a^calculated based on the data from (22); ^b^ fixed during fitting procedure

From this table, the drug-independent parameter values, i.e., *k*_*out*_, *k*_*deg*_, *t*_*lag*_, *k*_*in(0)*_, and *τ* were similar among all investigated groups despite the fact of using different vehicles and routes of administration. The value of *k*_*grow*_ was calculated based on the results of the previous study performed in Lewis rats (22) and fixed during iteration process. Rolipram demonstrated the highest inhibitory properties as indicated by the *IC*_*50*_ value, which was the lowest among the tested compounds. GRMS-55 exhibited ~26 and (±)-LSF ~100 times higher *IC*_*50*_ value than rolipram. However, the observed differences in relative paw swelling among the treatment groups were not different statistically at any of the time points measured. TNF-α serum levels in rats from groups treated with the investigated compounds and vehicle alone measured at the end of the experiment by ELISA were below the LOQ (12.5 pg mL^−1^). Additional data obtained, such as time courses of the mean relative body weights, clinical score values, and paw areas with the results of statistical analysis were presented in supplementary materials (Fig. 2S).

### Histological Evaluation

**Fig. 7:**
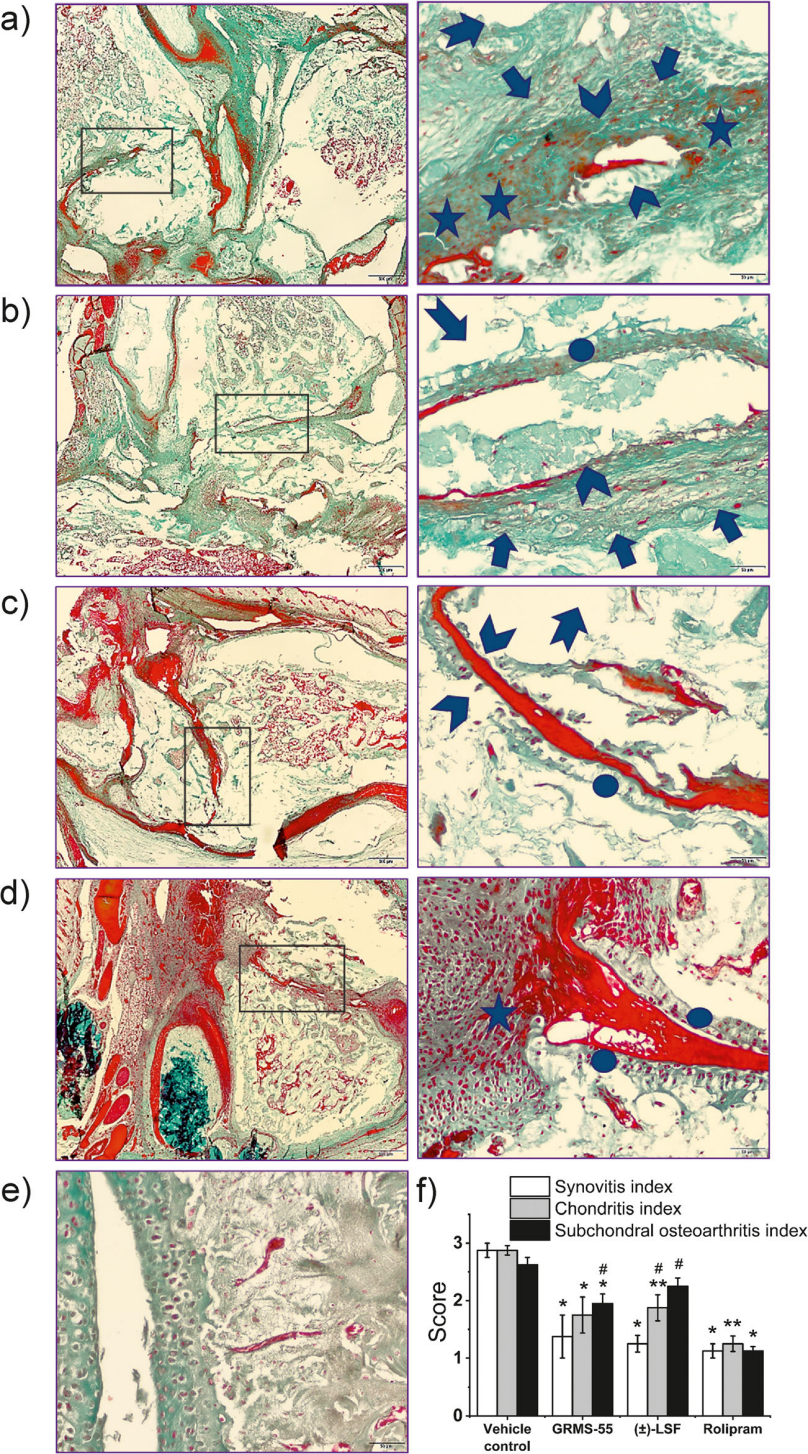
Histological examination of ankle cross-sections from arthritic rats treated with vehicle only (positive control) **a**, rats administered with GRMS-55 **b**, animals treated with (±)-LSF **c**, rats administered with rolipram **d**, and healthy animals not subjected to the immunization procedure **e**. On the left side, a low-power magnified view (objective lens 4x) of ankle joints is presented. On the right side, a high-power magnified view (objective lens 40x) of boxed area indicated on the left is shown. Synovitis and leucocytes infiltrating into synovial fluid spaces and synovium are shown as asterisks. Articular cartilage degeneration is depicted as arrow heads. Arrows represent subchondral plate thickening, whereas arrows with tails indicate trabeculae deterioration, cavitary lesions, and fibrovascular replacement of marrow spaces. Ellipses match regenerating articular cartilage. **f** Mean (±) standard error histological scores for three categories: synovitis, chondritis, and subchondral osteoarthritis assessed in ankle cross-sections from rats with CIA treated with vehicle only and the investigated compounds; *p < 0.05, **p < 0.01– compared to the vehicle control group, ^#^p < 0.05 – compared to the rolipram treated group (n = 4) Kruskal–Wallis with Dunn’s multiple comparison post-hoc test.

Histopathological assessment aimed to determine overall pattern of injury and the scoring focused on a grade of synovial inflammation and articular cartilage lesions associated with subchondral bone injury. Animals in positive control group (vehicle treated CIA rats) exhibited full range of pathological symptoms, such as severe synovial inflammation, cartilage degeneration revealed by its surface integrity loss, chondrocyte alteration (loss or hypercellurarity and/or cloning), and subchondral lesions: enormous subchondral plate thickening, trabeculae deterioration, bone marrow lesions (bone “pseudocysts” - cavitary lesion forming) frequently associated with fibrovascular replacement of marrow spaces (Fig. 7).

Figure 7 shows that administration with all studied compounds to CIA rats led to a significant amelioration of synovial inflammation and concurrent articular cartilage and subchondral bone remodeling in relation to the vehicle control, except (±)-LSF in the case of subchondral osteoarthritis. Treatment with rolipram led to a significant amelioration of these symptoms compared to vehicle control. The treatment with both GRMS-55 and (±)-LSF induced a significant alleviation of histopathological appearance of synovium compared to vehicle treated animals. In particular, synovial linning became thinner and cellularity of inflammatory cells sparse. After multiple administration of both GRMS-55 and (±)-LSF, chondritis and subchondral osteoarthritis were attenuated compared to vehicle control, except for (±)-LSF in the case of subchondral arthritis. Rolipram was more potent than GRMS-55 in alleviating subchondral osteoarthritis and more active than (±)-LSF in ameliorating chondritis and subchondral osteoarthritis. In contrast to the vehicle control group, structural healing changes, such as regenerating articular cartilage in rats treated with the tested compounds can be observed.

## Discussion

The aim of this study was to compare the pharmacological activity of the two PDE inhibitors, namely GRMS-55 and (±)-LSF. We demonstrated that PDE4B but not PDE4D blockage is essential to obtain a therapeutic effect in animal models of immune-mediated hepatitis, autoimmune rheumatoid arthritis, and endotoxemia. This observation indicates that compounds revealing strong to moderate PDE4B but not PDE4D inhibitory properties may constitute a better therapeutic option compared to pan-PDE4 inhibitors, which are burdened with gastrointestinal adverse effects arising from PDE4D inhibition. Furthermore, it has been indirectly demonstrated that a PDE7A inhibitory component may reinforce the therapeutic activity in animal models of immune-related disorders, however, the observed therapeutic effect seems to be mainly dependent on the potency of PDE4B inhibition. Finally, we developed PK/PD and PK/PD/DIS progression models, which may serve for further studies on anti-inflammatory and immune-regulatory properties of PDE inhibitors. Our results indicate that both GRMS-55 and (±)-LSF are interesting compounds for future pre-clinical and clinical studies on the treatment of immune-related disorders.

Due to the ubiquitous expression of different types and subtypes of PDEs in the human body and their involvement in a variety of signaling pathways, PDE inhibitors have emerged as a large group of drugs for the treatment of various disorders. However, despite the undeniable therapeutic potential of these compounds, some PDE inhibitors are burdened with certain adverse effects. For instance, potent PDE3 inhibitors demonstrate a strong inotropic effect and may induce life-threatening arrhythmias in people with heart rhythm disorders (23). In turn, potent PDE10A inhibitors have been shown to induce cataleptic-like effect in rodents (8). However, in clinical trials somnolence was the most commonly observed adverse effect in this group of compounds. PDE4 inhibitors induce gastrointestinal adverse effects, such as nausea and emesis. Therefore, they are often poorly tolerated by patients. The results of pre-clinical studies indicate that PDE4D inhibition is responsible for this type of adverse effects (15). GRMS-55 demonstrated a moderate and (±)-LSF weak activity against hrPDE3A, which indicates a limited possibility of occurrence of the side effects resulting from the inhibition of this type of PDE. Moreover, both compounds did not inhibit hrPDE4D at the concentrations tested. GRMS-55 exhibited only weak inhibitory properties against hrPDE10A. On the other hand, both compounds inhibited hrPDE4B, which is at least partially responsible for the therapeutic effect of these compounds. In addition, GRMS-55 is a potent hrPDE7A inhibitor. This feature may cause an additional anti-inflammatory and/or immunomodulatory effect, as dual PDE4/7 inhibitors demonstrated favorable properties compared to selective PDE4 inhibitors in a number of pre-clinical studies (2). These profiles of PDE inhibition of GRMS-55 and (±)-LSF seem to be very preferential compared to that of rolipram, which is a potent pan-selective PDE4 inhibitor similarly inhibiting both PDE4B and PDE4D subtypes. Due to the latter feature of this compound, it induced nausea and emesis in clinical trials, and therefore, despite the number of beneficial properties, it has not been registered as a drug so far (6). The results of PDE inhibitory activity assay performed in this study are in agreement with the results obtained in earlier studies, where vinopocetine (24), EHNA (25), milrinone (26), rolipram, BRL-50481 (27), and papaverine (8) demonstrated similar *IC*_*50*_ values against corresponding types of PDE. Only in the case of zaprinast, the estimated *IC*_*50*_ value was higher than that observed in the previous studies (28), probably due to the relatively high concentration of cGMP (5 μM) which was required in the PDE assay used in this study.

In the *in vitro* TNF-α inhibition assay, rolipram occurred to be the most potent molecule, even at a concentration of 50 μM. In contrast, GRMS-55 at the levels of 50 and 250 μM failed to inhibit TNF-α release compared to the control wells, while both were active *in vivo*. The possible reasons for the observed discrepancy may be a limited solubility of GRMS-55 in the assay medium or an interaction of GRMS-55 with other, than present in blood monocytes and lymphocytes, immune cells responsible for TNF-α release upon stimuli *in vivo*. For example, Kupffer cells or alveolar macrophages may release TNF-α upon LPS stimulation (29,30) and at the same time they may be more susceptible to anti-inflammatory activity of some PDE inhibitors. The observed phenomenon, although not elucidated yet, indicates some limitations of this widely used *in vitro* test for the screening of anti-inflammatory compounds. On the other hand, (±)-LSF and its metabolite – PTX exhibited comparable, dose-dependent inhibitory properties. However, even at a concentration of 250 μM these compounds acted significantly weaker than rolipram at a lower concentration. These results are in agreement with the earlier study on PDE inhibitors, where rolipram and other PDE4 inhibitor RP73401 (*IC*_*50PDE4*_ = 1 nM) exhibited a dose dependent activity in LPS-stimulated murine macrophages and human PBMC. The *IC*_*50*_ values were 2.7 nM for RP73401 and 59.3 nM for rolipram in murine macrophage cell line and 3.3 nM for RP73401 in PBMC. Furthermore, PTX also significantly inhibited TNF-α production in that study in RAW263.7 cells at concentrations of 500 and 1000 μM (31). Moreover, a correlation between suppressive function of PDE4 inhibitors on antigen-stimulated T cell proliferation and their inhibitory activity against PDE4A and PDE4B subtypes was observed, while no correlation was noted with respect to a PDE4D inhibitory activity. Similarly, the potency of inhibition of LPS-induced TNF-α release from human monocytes was significantly correlated with the inhibition of PDE4A (r = 0.903, *P* = 0.001) and PDE4B (r = 0.891, P = 0.001). In contrast, a correlation between the inhibitory activity on TNF-α release and PDE4D was not statistically significant (r = 0.600, *P* = 0.073) (1), which implies that an inhibition of PDE4B but not PDE4D is crucial to obtain the anti-inflammatory effect of PDE4 inhibitors.

ConA-induced hepatitis in mice is a widely used model of T cell mediated liver injury, which in many aspects closely resembles AIH – a severe human disorder that often leads to liver fibrosis and, in consequence, to liver failure. Therefore, this model has been frequently utilized in the search for new drugs and treatment options for AIH (32). In the course of ConA-induced hepatitis, similarly to AIH, T-cells and a wide range of cytokines are engaged in the disease process and serum transaminase activity is dramatically increased. The current conventional treatment of AIH is based on the administration of a corticosteroid in combination with azathioprine. Usually it must be continued lifelong, despite the fact of severe adverse effects of these drugs that often manifest. But even if treatment is implemented, some of the patients do not achieve complete remission. In these cases, progressive fibrosis of the liver leads to the liver failure, when the only remaining treatment option is the organ transplantation. TNF-α is considered as one of the crucial mediators of liver damage in AIH, thus, anti-TNF-α biological drugs have been used in difficult-to-treat patients suffering from this disorder (11). PDE inhibitors have been earlier successfully tested in the ConA-induced hepatitis model in mice. A selective PDE4 inhibitor – rolipram has been shown to ameliorate the outcomes of this disease. It decreased TNF-α, IFN-γ, and IL-4 levels and increased concentrations of an anti-inflammatory cytokine, IL-10. It also reduced plasma ALT and AST activities. Motapizone, a selective PDE3 inhibitor, exhibited similar properties to those of rolipram. However, it did not change IL-4 levels significantly at a range of doses tested (0.1–10 mg kg^−1^) (33). In the present study, similar observations were made as rolipram reduced levels of TNF-α and decreased AST and ALT activities in serum of mice. In another study, a PDE inhibitor with a strong activity against PDE7A (*IC*_*50*_ = 9 nM) has been investigated in the ConA-induced hepatitis model and it decreased ALT activity and TNF-α levels (34). Thus, it can be concluded that the observed effects of PDE inhibitors in ConA-induced hepatitis arise both from the inhibition of PDE4B and PDE7A. Moreover, as it has been shown in the previous studies, PDE3 inhibition can additionally support this effect. As expected, GRMS-55, which is a potent hrPDE7A and a moderate hrPDE3A/4B inhibitor, exhibited a strong therapeutic effect in ConA-induced hepatitis. On the other hand, (±)-LSF, which is a non-selective PDE inhibitor with weaker PDE3A, PDE4B, and PDE7A inhibitory properties, failed to significantly decrease the activity of transaminases at an IP dose of 50 mg kg^−1^, however, it decreased TNF-α concentration in mice serum. The observed discrepancy may arise from an additional activity of (±)-LSF, which demonstrates STAT4 inhibitory properties (35). It has been shown, STAT4 deficient mice exhibited an increased susceptibility to ConA in ConA-induced hepatitis (36). Thus, an inhibition of STAT4 by (±)-LSF could have led to the exacerbation of the disease.

Pharmacokinetics of (±)-LSF has been already deeply investigated in rats (20) but also in other species. The results of our experiments are in agreement with those from earlier studies on this compound in rats. Some observed differences could have arisen from the use of different strains and sexes of rats. Female Lewis rats displayed 3.3 times lower value of volume of (±)-LSF distribution compared to male Wistar rats. Moreover, in the earlier studies in male rodents, an existence of nonlinearity in the process of elimination of (±)-LSF was observed, while in the case of female Lewis rats the elimination was linear. A high bioavailability following SC dosing was confirmed in this study, indicating that this route of administration is the most appropriate for pre-clinical studies on (±)-LSF (20). In the present study, rolipram exhibited a fast absorption following IP administration and a quite fast elimination, as the elimination half-life of this compound was equal to 11 min in the case of male and 30 min in female rats. The results of earlier study indicate that this compound has the elimination half-life of 1–3 h depending on the species (37). GRMS-55 demonstrated a linear pharmacokinetics and a moderate bioavailability following IP dosing. It was quite quickly eliminated from rat plasma with elimination half-life values of 22 min in male and 21 min in female rats. However, a 2-compartment model was more relevant to describe pharmacokinetics of GRMS-55 in female rats, while a 1-compartment model was more suitable in the case of male rodents. The limitation of this study is that the effects of the rat strain and sex on pharmacokinetics of the investigated compounds were not evaluated, because different rat sexes of each strain (Wistar and Lewis) were used in the experiments. Therefore, it was not possible to assess the impact of these factors on the values of pharmacokinetic parameters of the tested compounds. Moreover, a more in-depth investigation of GRMS-55 pharmacokinetics is required to accurately determine the structural pharmacokinetic model for this compound. The results of the present study indicate that the pharmacokinetic model for GRMS-55 is highly dependent on the experimental data available as the concentration-time data were captured by different models (1- *vs* 2-compartment) depending on the groups of rats studied or the applied route of administration.

LPS-induced endotoxemia is a model of sepsis used to evaluate anti-inflammatory agents, as LPS is a crucial trigger of inflammation in bacterial infection and sepsis. However, this test may be also used to evaluate the TNF-α reducing potential of investigated compounds. PDE inhibitors have been previously tested as anti-inflammatory agents in LPS-induced sepsis in rodents. Cilostazol, a PDE3 selective inhibitor, has been shown to improve the survival of mice in a model of LPS-induced septic shock. TNF-α, IL-1β, and IL-6 levels were significantly decreased in animals pre-treated with this compound at a dose range of 1–5 mg kg^−1^. Moreover, rolipram and other PDE4 inhibitor, CP-77059 reduced serum TNF-α levels and mortality in the LPS-induced septic shock model. PK/PD modeling is rather rarely used in assessing the potency of anti-inflammatory drugs in endotoxemia models, although, there have already been some attempts to use it to evaluate the potency of some compounds, including (±)-LSF and PTX (19), to assess the nature and the potency of interactions between drugs (38), but also to elucidate the mechanism of anti-inflammatory activity (39). In a mice model of endotoxemia, R-(−)-LSF as well as PTX have demonstrated strong anti-inflammatory properties. *IC*_*50*_ value for the inhibition of TNF-α release estimated for R-(−)-LSF was 1.61 and 0.47 mg L^−1^ for PTX (19). In turn, in the experiment in rats using cAMP as a marker of pharmacological response, the *IC*_*50*_ value for the the inhibition of cAMP degradation was 3.43 for (±)-LSF and 4.48 mg L^−1^ for PTX. In the latter study, an additive interaction between them was assumed, and a modified indirect response model was utilized to assess the potency of these compounds. In the present study, similarly to the earlier experiments in mice, we used TNF-α plasma levels as a marker of pharmacological response. (±)-LSF demonstrated the *IC*_*50*_ value for the inhibition of TNF-α release equal to 5.8, while for PTX it was 14.0 mg L^−1^, so (±)-LSF was ~2.4 times stronger than PTX. GRMS-55 in this test exhibited quite strong inhibitory properties expressed as *IC*_*50*_ equal to ~1.1 mg L^−1^, while rolipram was the most potent inhibitor of TNF-α release demonstrating almost 3 times higher activity than GRMS-55 and approx. 16 and 39 times higher potency than those of (±)-LSF and PTX, respectively. The results of this experiment indicate that the inhibition of TNF-α release is associated with PDE4B inhibitory activity of each compound, while PDE4D inhibition is not necessary to obtain the therapeutic effect. The simultaneous inhibition of other PDE subtypes, such as PDE7A, PDE3A, or PDE1B may possibly augment the therapeutic effect in LPS-induced endotoxemia, as GRMS-55, which demonstrates strong PDE1B and PDE7A and moderate PDE3A inhibitory properties, was ~5.5 more potent than (±)-LSF in inhibiting TNF-α release, while it is only ~2 times more potent as a PDE4B inhibitor.

The PK/PD model developed in this study to describe TNF-α changes following LPS administration to rats is a modified indirect response model (19) with implemented transit compartments reflecting the signal transduction process that takes place from the moment of LPS administration to an increase in levels of TNF-α in the rat blood. Individual transit compartments correspond to subsequent stages leading to TNF-α release, such as binding of LPS to TLR4 receptor, subsequent signal transmission, TNF-α gene transcription, TNF-α protein formation and its release from immune cells into the blood. The τ parameter value is related to the duration of these stages. Whereas the *k*_*in*_ value is the time limited zero-order rate constant of production of a precursor occurring on the signaling pathway leading to TNF-α release. The assumption of this model is that the tested compounds act by inhibiting the inflammatory mediator called the precursor. This is in agreement with the mechanism of action of PDE4B inhibitors, as these compounds inhibit the synthesis of TNF-α mRNA that may be considered a precursor of TNF-α. Thus, this model closely resembles the mechanism of development of LPS-induced endotoxemia and the mechanism of action of the tested compounds. Furthermore, the number of estimated parameters in the model developed in this study is reduced. By using a signal transduction, it was possible to eliminate one lag time parameter that was used in the previous studies (19), which was rather an artificial parameter describing the delay in the observed effect of the test compounds in relation to they appearance in blood.

RA is a chronic and debilitating autoimmune disorder leading to disability and a significant deterioration of the quality of life. CIA in rat is the most recognized and clinically relevant animal model of RA, characterized by the destruction of bones and cartilage, the presence of autoantibodies, and an involvement of TNF-α as the main mediator leading to the development of this disease. Some PDE inhibitors have been already tested in CIA. Selective PDE4 inhibitors, such as rolipram and apremilast have been demonstrated to alleviate the disease development and progression. Rolipram exhibited a preventive role in the development of CIA by decreasing a clinical score and reduced TNF-α and IFN-γ mRNA synthesis in cells isolated from draining lymph nodes of rats with CIA (40). A dual PDE4/7 inhibitor – YM-393059 ameliorated the clinical signs of the CIA in mice by suppressing TNF-α and IL-1β production and suppressing humoral immune response. Interestingly, this compound exhibited a reduced emetogenic potential compared to that of a selective PDE4 inhibitor (41). The results of the present study confirm the therapeutic potential of selective PDE4 inhibitors, as the most potent PDE4 inhibitor among the investigated compounds – rolipram exhibited the strongest therapeutic potential in CIA. The PK/PD analysis performed in this study allowed for the quantitative evaluation of the potency and comparison of each investigated compound by correlating the collected concentration-effect data. The *IC*_*50*_ values for an inhibition of the production component (*k*_*in*_*(t)*), which represents the disease progression, were estimated for the three investigated compounds, namely rolipram, GRMS-55, and (±)-LSF. GRMS-55 was ~26 times less potent than the selective PDE4 inhibitor – rolipram, but ~4 times stronger in inhibiting the disease progression than (±)-LSF. These results imply that probably PDE4B inhibition is mainly responsible for the antiarthritic potential of the investigated compounds, but PDE7A inhibition may also play a role in the treatment process, as GRMS-55 was ~4 times stronger than (±)-LSF, which was only ~2 times weaker as a PDE4B inhibitor *in vitro* than GRMS-55. Nevertheless, further studies using selective PDE7 inhibitor in CIA model are necessary to elucidate the possible role of selective PDE7 inhibition in the treatment of immune-mediated arthritis. With no doubt, it has been demonstrated that an inhibition of PDE4D is not necessary to achieve a therapeutic effect in this disease model. In general, it can be concluded that the lower is the value of *IC*_*50*_ for a particular compound with respect to PDE4B, the higher is the potency of this compound in reducing progression of CIA. GRMS-55 and (±)-LSF, which have higher *IC*_*50*_ values against hrPDE4B, demonstrated a weaker antiarthritic potential than rolipram in this study. However, it should be kept in mind that they may be used at higher doses due to their favorable safety profiles, which may make up for the lower potency.

In this study, we modified the PK/PD/DIS progression model developed earlier by Earp *et al.* (18). A relative size of each rat paw was normalized to the size of the same paw at the day of the first immunization. Thus, paw size at day 0 was fixed to 1, and initial conditions for each transit compartment were fixed to 1 as well. The natural paw growth rate was calculated based on the study performed in healthy female Lewis rats (21) and fixed. *I*_*max*_ values were fixed to 1. The transit compartments were implemented to illustrate the delay in the onset of paw swelling from the time of immunization and to illustrate the delay of the effect occurrence following the administration of the drug. The use of transduction compartments was beneficial, as it made the model more relevant to the mechanism of the CIA development. Following collagen type II administration, the binding between the peptide and the MHC molecules occurs, the specific antibodies are produced, T and B cells are recruited, and the production of cytokines, such as TNF-α, which is also divided into several stages (transcription and translation) occurs. Therefore, undoubtedly, the pathogenesis of CIA is a multistep process initiated by the administration of collagen emulsion, the final effect of which is the appearance of visible symptoms of the disease, such as swelling and redness of joints. Moreover, the PDE inhibitors, which exert the therapeutic effect, probably do not act directly on the production of swelling of the paw, as it was assumed in the previously developed PK/PD/DIS models for other compounds, but they may interact with various factors preceding the onset of swelling, such as an increased gene expression of pro-inflammatory cytokines or proliferation of lymphocytes. Thus, the model developed in this study seems to more closely resemble both the pathogenesis of the disease and the mechanism of action of the investigated compounds. Nevertheless, previously developed models might have been more relevant in studying other types of drugs, such as biologics, which exert their activity at other stages of the disease development and may interact with other targets. Different variants of input components have been presented in the literature in PK/PD/DIS progression models to describe a sharp increase of edema after arthritis onset and a natural remission of the disease in rat CIA. Among others, a production rate was used by Earp *et al.* (18), as a function of time starting at the disease onset and being dependent upon the degree of swelling. A combination of transduction and indirect response models has been also previously utilized to this end (42). In that study, 19 transduction compartments were employed to describe the time delay to sharp increase of paw size and 5 more to account for the time delay of natural remission after disease onset. Moreover, a nonlinear negative feedback mechanism was utilized to capture the natural remission. Unfortunately, all previously developed and tested in this study models resulted in considerable imprecision of estimated parameters. Therefore, in the model developed in this study, similarly as in the previous work of Lon *et al.* (43), we have employed the empirical input, where the *k*_*deg*_ is the first-order rate constant that represents a decline in the zero-order rate *k*_*in*_*(t)* and accounts for the natural remission of arthritis after disease onset. In addition, we utilized transduction compartments accounting for delay between the disease onset and swelling of paws. This way of the defining the production component allowed for a satisfactory fit of the model to the experimental data and an effective comparison of the activity of the tested compounds.

The results of histological examination of paw cross-sections from rats with CIA are in line with the results of earlier experiments in rodents with CIA, where symptoms such as subchondral bone destruction, cartilage disruption, or synovitis were observed. These manifestations were alleviated by the administration of other, than used in this study, PDE4 inhibitors, such as apremilast (44). The statistical analysis of histological scores confirmed that rolipram exhibited the strongest activity in ameliorating the abovementioned symptoms of CIA, while GRMS-55 as well as (±)-LSF exhibited weaker but also significant antiarthritic properties.

## Conclusion

In conclusion, the investigated PDE inhibitors, namely GRMS-55 and (±)-LSF exhibit favorable PDE inhibitory profiles, which are a good prognosis for their therapeutic effectiveness and safety of use. GRMS-55 is a strong hrPDE7A and hrPDE1B inhibitor with moderate hrPDE4B and hrPDE3A inhibitory properties. On the other hand, (±)-LSF is a moderate hrPDE3A and hrPDE4B inhibitor. Both compounds do not demonstrate activity against hrPDE4D, an inhibition of which is considered to be responsible for adverse effects, such as nausea and emesis. It has been demonstrated that PDE4D inhibitory activity is not essential to obtain therapeutic effect in all tested animal models of immune-related diseases. In contrast, the potency of PDE4B inhibition appears to be related to anti-inflammatory properties of the investigated compounds. GRMS-55 ameliorated ConA-induced hepatitis in mice by inhibiting NF-α secretion and reducing the activity of transaminases. On the other hand, (±)-LSF decreased TNF-α levels but failed to reduce the activity of transaminases. This failure might have arisen from STAT4 inhibitory properties of (±)-LSF. Both compounds exhibit a moderate activity in LPS-induced endotoxemia model and CIA, compared to that of a potent pan-PDE4 inhibitor – rolipram, which most strongly inhibited CIA progression and TNF-α plasma levels in LPS-induced endotoxemia. Furthermore, all compounds tested alleviated histological symptoms of arthritis, however, rolipram demonstrated the strongest potency in reducing subchondral osteoarthritis, while (±)-LSF failed to significantly diminish this manifestation. The inhibition of PDE7A seems to display an additional anti-inflammatory effect, as a strong inhibitor of PDE7A – GRMS-55 demonstrated ~5.5 and ~4.0 times lower *IC*_*50*_ values in LPS-induced endotoxemia and CIA, respectively, than (±)-LSF, which is only a ~2.0 times weaker PDE4B inhibitor than GRMS-55. The developed in this study PK/PD and PK/PD/DIS progression models may be used to design future pre-clinical studies and to assess the potency of new compounds for the treatment of RA and other inflammatory disorders. GRMS-55 and (±)-LSF seem to be promising candidates for further pre-clinical and clinical studies of immune-related diseases.

## Electronic Supplementary Material

ESM 1 (DOCX 535 kb)

## Abbreviations

AIH: Autoimmune hepatitis
ALT: Alanine aminotransferase
AST: Aspartate aminotransferase
cAMP: 3′,5′-cyclic adenosine monophosphate
cGMP: 3′,5′-cyclic guanosine monophosphate
CIA: Collagen induced arthritis
ConA: Concanavalin A
DMSO: Dimethyl sulfoxide
HPLC: High performance liquid chromatography
hrPDE: Human recombinant phosphodiesterase
IFN-γ: Interferon γ
IL: Interleukin
LPS: Lipopolysaccharide
(±)-LSF: Lisofylline
MHC: Major histocompatibility complex
NCA: Non-compartmental analysis
PBMC: Peripheral blood mononuclear cells
PDE: Phosphodiesterase
PEG: Polyethylene glycol
PK/PD: Pharmacokinetic/pharmacodynamic
PK/PD/DIS: Pharmacokinetic/pharmacodynamic/disease progression
PTX: Pentoxifylline
RA: Rheumatoid arthritis
STAT4: Signal transducer and activator of transcription 4
TLR4: Toll-like receptor 4
TNF-α: Tumor necrosis factor α
