# Supplementary material for: Comparative Assessment of the New PDE7 Inhibitor – GRMS-55 and Lisofylline in Animal Models of Immune-Related Disorders: A PK/PD Modeling Approach
Source: Pharm Res. 2020 Jan 2;37(2):19. doi: 10.1007/s11095-019-2727-z (PMC6940354; doi:10.1007/s11095-019-2727-z)
Supplement: Supplementary file 1 — (DOCX 535 kb) [file 11095_2019_2727_MOESM1_ESM.docx]

Comparative assessment of the new PDE7 inhibitor - GRMS-55 and lisofylline in animal models of immune-related disorders: A PK/PD modeling approach

Artur Świerczek^1^, Krzysztof Pociecha^1^, Marietta Ślusarczyk^2^, Grażyna Chłoń-Rzepa^2^, Sebastian Baś^3^, Jacek Mlynarski^3a^, Krzysztof Więckowski^4^, Monika Zadrożna^5^, Barbara Nowak^5^, and Elżbieta Wyska^1*^

^1^Department of Pharmacokinetics and Physical Pharmacy, Jagiellonian University Medical College, 9 Medyczna Street, 30-688 Kraków, Poland

^2^Department of Medicinal Chemistry, Jagiellonian University Medical College, 9 Medyczna Street, 30-688 Kraków, Poland

^3^Faculty of Chemistry, Jagiellonian University, Gronostajowa 2, 30-387 Kraków, Poland

^4^Department of Organic Chemistry, Jagiellonian University Medical College, 9 Medyczna Street, 30-688 Kraków, Poland

^5^Department of Cytobiology, Jagiellonian University Medical College, 9 Medyczna Street, 30-688 Kraków, Poland

Current address: Institute of Organic Chemistry, Polish Academy of Sciences, Kasprzaka 44/52, Warsaw, Poland

Running head: PK/PD of PDE inhibitors in immune-related diseases

*Corresponding author:

Elżbieta Wyska

Department of Pharmacokinetics and Physical Pharmacy,

Jagiellonian University Medical College,

9 Medyczna Street, 30-688 Kraków, Poland

phone: +48(12) 6205720

fax: +48(12) 6205730,

e-mail: [mfwyska@cyf-kr.edu.pl](mailto:mfwyska@cyf-kr.edu.pl)

Supplementary material

# Additional method description

## PDE assay procedure

One and a half μL aliquots of PDE-Glo reaction buffer containing appropriate amounts of human recombinant PDE (hrPDE) 1B, 2A, 3A, 4B, 4D, 5A, 7A, or 10A (SignalChem, Richmond, Canada) were placed into separate wells of a 384-well plate (Thermo Scientific, USA). The investigated compounds were initially dissolved in DMSO and then a serial dilution with DMSO was performed. Each dilution was mixed with PDE-Glo reaction buffer at a ratio of 1:5 (v/v). Then, 1 μL of the solution containing a PDE inhibitor and 2.5 μL of the substrate solution were added to each well. cGMP was used as a substrate in the case of hrPDE2A and hrPDE5A, while cAMP for other types of hrPDEs. The samples were incubated for 10 min at 30°C. Subsequently, 2.5 μL volumes of PDE-Glo Termination Buffer and PDE-Glo Detection Solution were added to each well and after 20 min of incubation at room temperature, 10 μL of Kinase-Glo Reagent was added to each well. The luminescence of each sample was measured by a microplate reader POLARstar Omega (BMG LABTECH, Germany).

## Determination of GRMS-55 and rolipram plasma levels

To determine GRMS-55 and rolipram in rat plasma, 10 μL of 4-(8-((Furan-2-ylmethyl)amino)-1,3-dimethyl-2,6-dioxo-2,3,6,7-tetrahydro-1*H*-purin-7-yl)-N’-(2-hydroxybenzylidene)butanehydrazide or temazepam solutions as internal standards (IS) for GRMS-55 and rolipram, respectively, were added to 100 μL of plasma samples (or to 100 μL of drug-free plasma samples spiked with a standard solution of one of the investigated compounds). The samples containing GRMS-55 were acidified with 20 μL of 1 M HCl acid solution and those containing rolipram were alkalized with 20 μL of 4 M NaOH solution. Then, the samples were extracted with 1 mL of dichloromethane for 20 min using a VXR Vibrax shaker (IKA, Germany). Subsequently, all tubes were centrifuged (10 000×g, 8 min) using EBA 12 R centrifuge (Hettich, Germany). Organic layers were collected and transferred to clear propylene tubes and evaporated in water bath under a gentle stream of nitrogen at 37°C. Dry residues were then dissolved in 100 μL of mobile phase and subjected to the HPLC analysis. The HPLC system (LaChrom Elite, Merck-Hitachi, Germany) consisted of an L-2130 pump, an L-2200 autosampler, and an L-2420 UV-VIS detector. EZChrome Elite v. 3.2 (Merck-Hitachi, Germany) computer program was used for data acquisition and integration. The separation of GRMS-55 was performed using a LiChrospher 100 RP-18 (250×4 mm) column with a particle size of 5 μm protected with a LiChroCART (4×4 mm, RP-18) guard column (Merck, Germany). Rolipram was separated using a LiChrospher RP-18 (125 × 4.6 mm) column with a particle size of 3 μm coupled with a guard column (10 × 4.6 mm) with the same packing material (Supelco , Germany). The mobile phase used for the analysis of GRMS-55 consisted of acetonitrile and 20 mM aqueous solution of KH_2_PO_4_ (pH = 4.5) mixed at a ratio of 55:45 (v/v) and pumped at a flow rate of 1 mL min^−1^, while the mobile phase used in the analysis of samples containing rolipram was composed of acetonitrile and water mixed at a ratio of 35:65 (v/v) and pumped at a flow rate of 0.7 mL min^−1^. Analytical wavelengths were set to 209 nm for GRMS-55 and 204 nm for rolipram analyzes. The separation of both investigated compounds was performed at ambient temperature. In these conditions, the retention times of rolipram and temazepam were 10.5 and 15.6 min, respectively. In turn, the retention time of GRMS-55 was 9.2 min. and that of its IS was 5.3 min.

## Sample preparation for histological evaluation

The hind paws were cut about 0.5 cm above the rat joint. Then the joints were fixed in 4% buffered formalin for one week, decalcified, dehydrated in ethanol solutions of increasing concentrations, and embedded in the paraffin blocks. Subsequently, paws were serially cut at 6-μm thickness using a rotational microtome Leica RM 2145 type and stained with Masson's Trichrome. Digital images were taken using a computerized digital camera (Olympus UC90, Olympus).

## Body weight and clinical score assessment

Body weight and paw size were measured, and clinical score was assessed at days 0, 5, 7, 11, 15, 18, 20, 22, 26, 30, 33, and 36 after the first immunization. Body weight of each rat was normalized to the body weight of the same rat at the day of the first immunization. Disease progression was evaluated by a qualitative clinical score (CS) as described elsewhere (1), where 0 – no symptoms of the disease, such as redness or swelling, 1 – mild, but definite redness and swelling, 2 – moderate redness and swelling, 3 – severe redness and swelling, and 4 – maximally inflamed limbs.

# Supplementary results

**PDE inhibitory activities of studied compounds**

**Fig. 1S** shows the representative graphs illustrating PDE inhibitory properties and fittings of the pharmacodynamic model (Eq. 1) to the concentration-effect data of each investigated and reference compound.


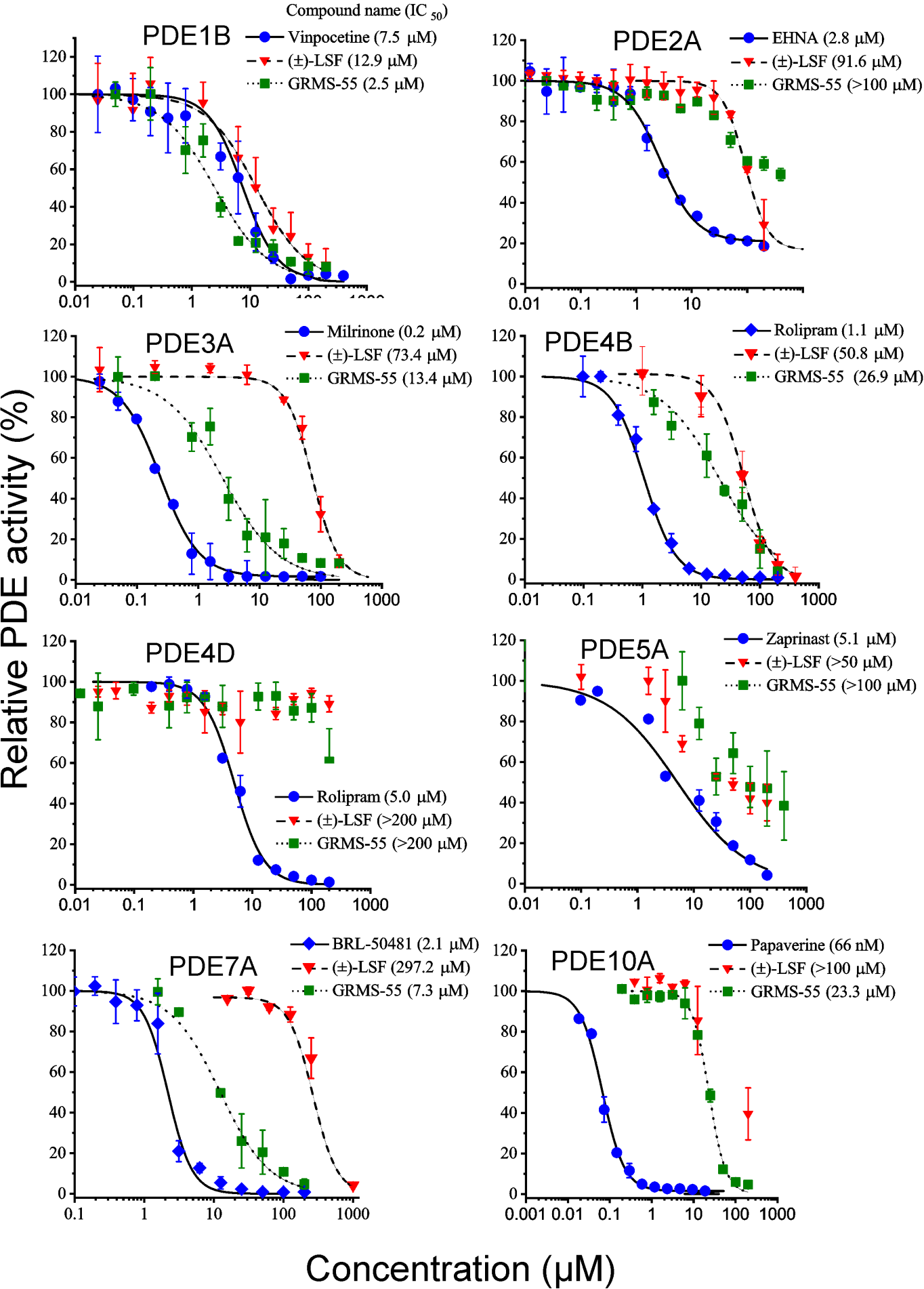


**Fig. 1S** Mean (±SD) measured (symbols) and predicted by the pharmacodynamic model (lines) relative activities of the investigated and reference compounds at various concentrations (n=4).

# Body weight and clinical score in rats with CIA

Time courses of the mean relative body weights, clinical score values, and paw areas were illustrated in **Fig. 2S**.





**Fig. 2S** Time course of mean (±SD) observed relative rat weights (a) and (b), mean clinical score factor values (±SD) (c) and (d) (*p<0.05, **p<0.01, Mann-Whitney U tests or Kruskal–Wallis test, compared to the control group), and paw areas (e) and (f) (*p<0.05, **p<0.01, Student t-test or a one-way ANOVA with Tukey HSD post-hoc test, compared to the control group) following multiple administration of GRMS-55, (±)-LSF, rolipram, or vehicle alone to rats with experimentally induced arthritis

As is shown in the graphs, in the period from the day of the first immunization to the 11^th^ day, a gradual increase in body weight of rats was observed, while no symptoms of the disease, such as swelling or redness of the skin around the joints, in most of the study and control groups were evident. From the 11^th^ day post the first immunization, a rapid decrease in body weight and a sharp increase in clinical score and paw section areas in all groups occurred, which reached peak values at the day 20^th^. After that day the clinical score and paw swelling gradually decreased in most groups, on the other hand, body weight increased. However, in the groups treated with rolipram, GRMS-55, and (±)-LSF the initial decrease in clinical score and paw edema was sharper than in control groups, while after 26^th^ day post the first immunization the decrease was more gradual. In the case of (±)-LSF a temporary increase in clinical score between 26^th^ and 30^th^ day was observed, while in the rolipram treated group at the same period a temporary increase in paw edema was noted. Paw areas were significantly lower in all study groups administered with investigated PDE inhibitors compared to the control groups at all measurements after 20^th^ day. In turn, clinical score values were significantly lower in the case of rolipram-treated group at all assessments post 20^th^ day and at 26, 33, and 36 day in the case of (±)-LSF-treated group. In contrast, GRMS-55 did not decreased significantly clinical score values of arthritic rats compared to the control group administered with vehicle alone. Moreover, there were no significant differences between clinical score values and paw areas in rolipram and GRMS-55 treated groups with the exception of paw area values at 26 day post the first immunization. There were no significant differences in body weight among the study and control groups throughout the experiment.

1. Protocol for the Successful Induction of Collagen-Induced Arthritis (CIA) in Rats. 2015.
